# Exploring the potential of the model cyanobacterium *Synechocystis* PCC 6803 for the photosynthetic production of various high-value terpenes

**DOI:** 10.1186/s13068-022-02211-0

**Published:** 2022-10-14

**Authors:** Victoire Blanc-Garin, Célia Chenebault, Encarnación Diaz-Santos, Marine Vincent, Jean-François Sassi, Corinne Cassier-Chauvat, Franck Chauvat

**Affiliations:** 1 Université Paris-Saclay, CEA, CNRS, Institute for Integrative Biology of the Cell (I2BC), 91198 Gif‐sur‐Yvette, France; 2 Commissariat À L’énergie Atomique Et Aux Énergies Alternatives (CEA), Centre de Cadarache, 13108 St Paul Lez Durance, France

**Keywords:** Cyanobacteria, Terpene diversity, Nitrogen sources (nitrate, urea), Genetic engineering, Production stability

## Abstract

**Background:**

The robust model cyanobacterium *Synechocystis* PCC 6803 is increasingly explored for its potential to use solar energy, water and atmospheric CO_2_ for the carbon-neutral production of terpenes, the high-value chemicals that can be used for the production of drugs, flavors, fragrances and biofuels. However, as terpenes are chemically diverse, it is extremely difficult to predict whether *Synechocystis* is a suitable chassis for the photosynthetic production of various terpenes or only a few of them.

**Results:**

We have performed the first-time engineering and comparative analysis of the best-studied cyanobacterium *Synechocystis* PCC 6803 for the photosynthetic production of five chemically diverse high-value terpenes: two monoterpenes (C_10_H_16_) limonene (cyclic molecule) and pinene (bicyclic), and three sesquiterpenes (C_15_H_24_) bisabolene (cyclic), farnesene (linear) and santalene (cyclic). All terpene producers appeared to grow well and to be genetically stable, as shown by the absence of changes in their production levels during the 5–9-month periods of their sub-cultivation under photoautotrophic conditions). We also found that *Synechocystis* PCC 6803 can efficiently and stably produce farnesene and santalene, which had never been produced before by this model organism or any other cyanobacteria, respectively. Similar production levels were observed for cells growing on nitrate (the standard nitrogen source for cyanobacteria) or urea (cheaper than nitrate). Furthermore, higher levels of farnesene were produced by cloning the heterologous farnesene synthase gene in a RSF1010-derived replicating plasmid as compared to the well-used slr0168 neutral cloning site of the chromosome.

**Conclusions:**

Altogether, the present results indicate that *Synechocystis* PCC 6803 is better suited to produce sesquiterpenes (particularly farnesene, the most highly produced terpene of this study) than monoterpenes (especially pinene).

**Supplementary Information:**

The online version contains supplementary material available at 10.1186/s13068-022-02211-0.

## Background

Terpenes are a widely diverse family of natural products with the formula (C_5_H_8_)_n_ that are often produced by plants. They can act in photosynthesis (chlorophyll, carotenoids and plastoquinone) and respiration (plastoquinone and ubiquinone), and/or emit floral scents that can attract pollinators or protect plants against pathogens. Many terpenes, such as bisabolene, farnesene, limonene, pinene and santalene can be used as platform compounds of interests for pharmaceutical, nutraceutical, cosmetic, and biofuel industries [1–3].

**Fig. 1:**
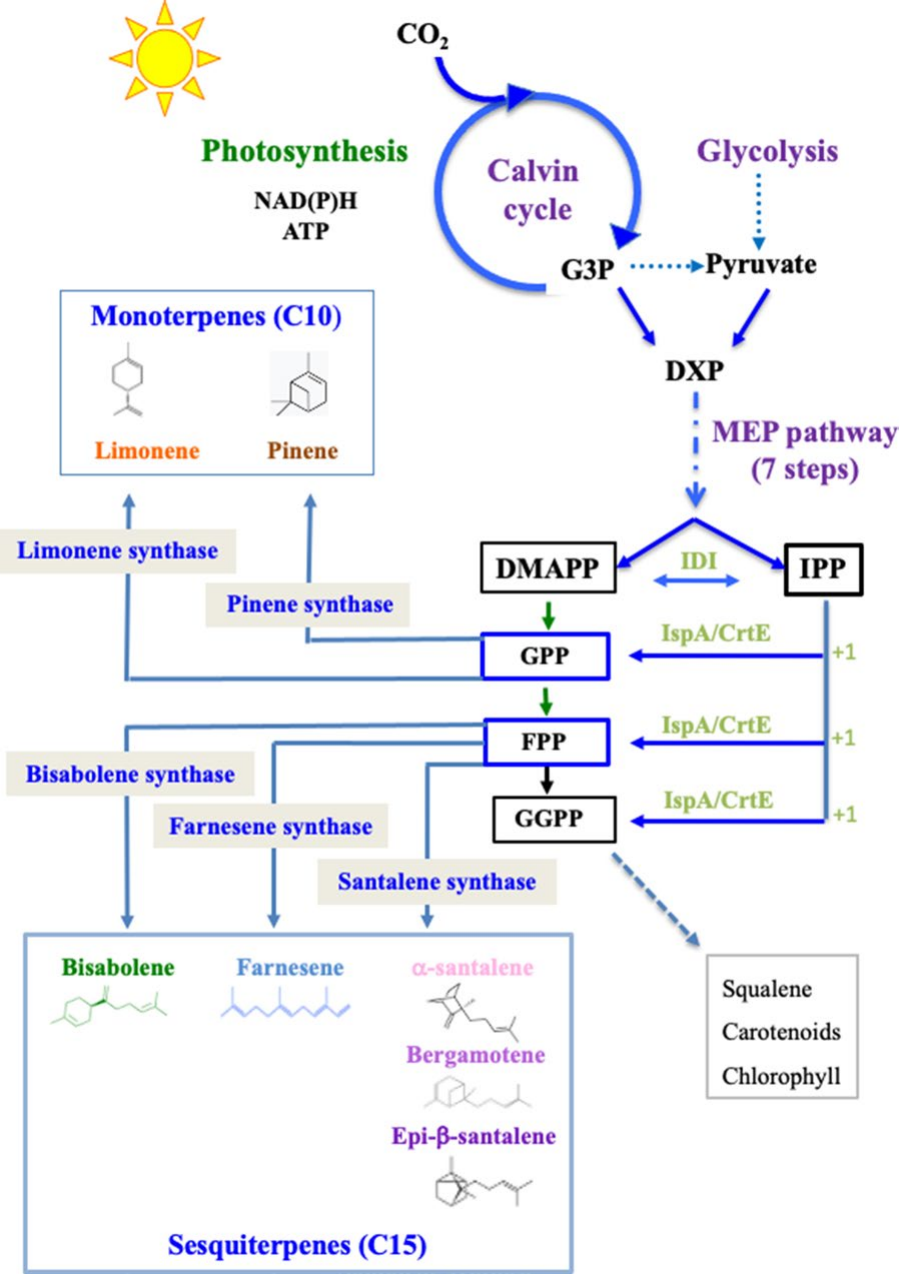
Schematic representation of the metabolic pathway of terpene biosynthesis from CO_2_. DMAPP: dimethylallyl pyrophosphate; DXP: 1-deoxy-d-xylulose-5-phosphate. G3P: glyceraldehyde 3 phosphate; IPP: isopentenyl pyrophosphate; GPP: geranyl pyrophosphate; FPP: farnesyl pyrophosphate; GGPP: geranylgeranyl pyrophosphate; MEP: methylerythritol 4-phosphate; IDI: isopentenyl-diphosphate; IspA/CrtE: isoprenyl diphosphate synthase

Terpenes are categorized based on the number of five-carbon units: hemiterpenes (C5), monoterpenes (C10), sesquiterpenes (C15), diterpenes (C20), triterpenes (C30), tetraterpenes (C40), and polyterpenes (≥ C45). They are all derived from the same five-carbon building blocks, isopentenyl pyrophosphate (IPP) and dimethylallyl pyrophosphate (DMAPP) (Fig. 1). IPP and DMAPP are condensed to form the C10 geranyl pyrophosphate (GPP), the precursor of monoterpenes. Consecutive addition of IPP units on GPP forms farnesyl pyrophosphate (FPP) and geranylgeranyl pyrophosphate (GGPP), the precursors of sesquiterpenes and diterpenes, respectively.

Because terpenes-producing plants are overexploited, such as *Santalum album* for sandalwood oil production [3], cyanobacteria, the oxygen-evolving photosynthetic prokaryotes, can be used for the sustainable production of terpenes from plentiful natural resources: solar energy, water, CO_2_ and a few minerals [2]. Cyanobacteria are robust organisms that colonize most water environments, where they outcompete most other microorganisms, thereby facilitating mass cultivations. They efficiently capture solar energy to fix a huge amount of atmospheric CO_2_ into a huge energy-dense biomass [4, 5] and they tolerate high CO_2_-containing (≥ 50%) gas [2]. Furthermore, they possess the methylerythritol-4-phosphate (MEP) pathway (Fig. 1) that utilizes glyceraldehyde-3-phosphate and pyruvate to produce GPP and FPP, which can be transformed into mono- and sesquiterpenes, providing synthetic genes encoding plant terpene synthases adapted to the cyanobacterial codon usage are introduced and expressed in cyanobacteria [2, 6]. The best-studied unicellular cyanobacterium *Synechocystis* PCC 6803 (hereafter *Synechocystis*) is particularly attractive for this purpose, in having good genetic tools [7]. Furthermore, *Synechocystis* is resistant to salt (euryhaline) and capable to grow on various nitrogen sources, such as nitrate and urea [8] which often pollute natural waters [9]. Moreover, *Synechocystis* has other biotechnological interests in producing biodegradable bioplastics (poly-hydroxybutyrate) and exopolysaccharides [4]. A few proof-of-concept studies carried out in different laboratories showed that *Synechocystis* can be engineered for the production of limonene [10, 11], pinene (very-weak production) [12] and bisabolene [13–15]. These terpene producers were generated by cloning codon-adapted terpene synthase genes, expressed from various promoters, in the chromosome or an autonomously replicating plasmid of either the wild-type strain or its glucose-tolerant derivative that were grown under different conditions. From such few and different studies, it is extremely difficult to predict whether *Synechocystis* is a suitable chassis for the production of many, chemically diverse, terpenes or only a few of them. To our knowledge, a single study [16] reported the engineering and comparative analysis of a cyanobacterium, i.e.*, Synechococcus* PCC 7002, distantly related to *Synechocystis*, for the (low-level) production of only two terpenes: limonene (∼4 mg L^−1^) and α-bisabolene (∼0.6 mg L^−1^).

**Fig. 2:**
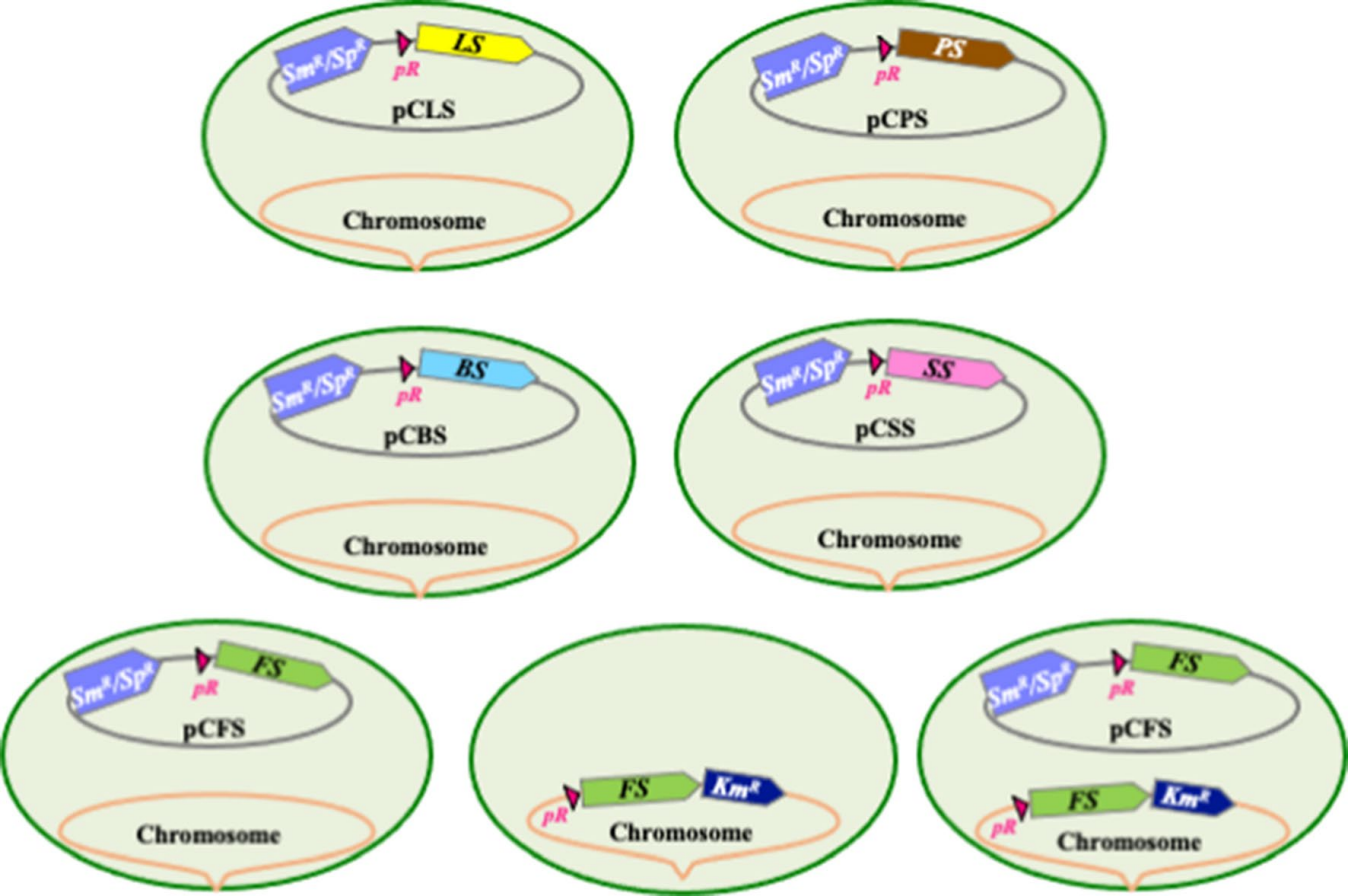
Schematic representation of the *Synechocystis* PCC 6803 strains engineered in this study. *Synechocystis* cells are shown as green oval shapes representing their chromosome (orange line) arbitrarily showed as attached to the cell membrane to distinguish it from the pC plasmids. Most strains harbor a derivative of the Sm^R^/Sp^R^ replicative plasmid vector pC (grey line) expressing a terpene-synthase encoding gene from the strong λ phage *pR* promoter (red triangle). These plasmids are: pCLS (limonene synthase, yellow arrow), pCPS, (pinene synthase, brown arrow), pCBS (bisabolene synthase, blue arrow), pCSS (santalene synthase, pink arrow) and pCFS (farnesene synthase, green arrow). Note that two other strains express the same *pR*-FS gene cassette from their chromosome (precisely from the slr0168 site, where it was introduced along with the Km^R^ marker gene). They served for the comparison of farnesene production driven by the *pR*-FS gene expressed from either of both the pCFS plasmid and the chromosome

**Fig. 3:**
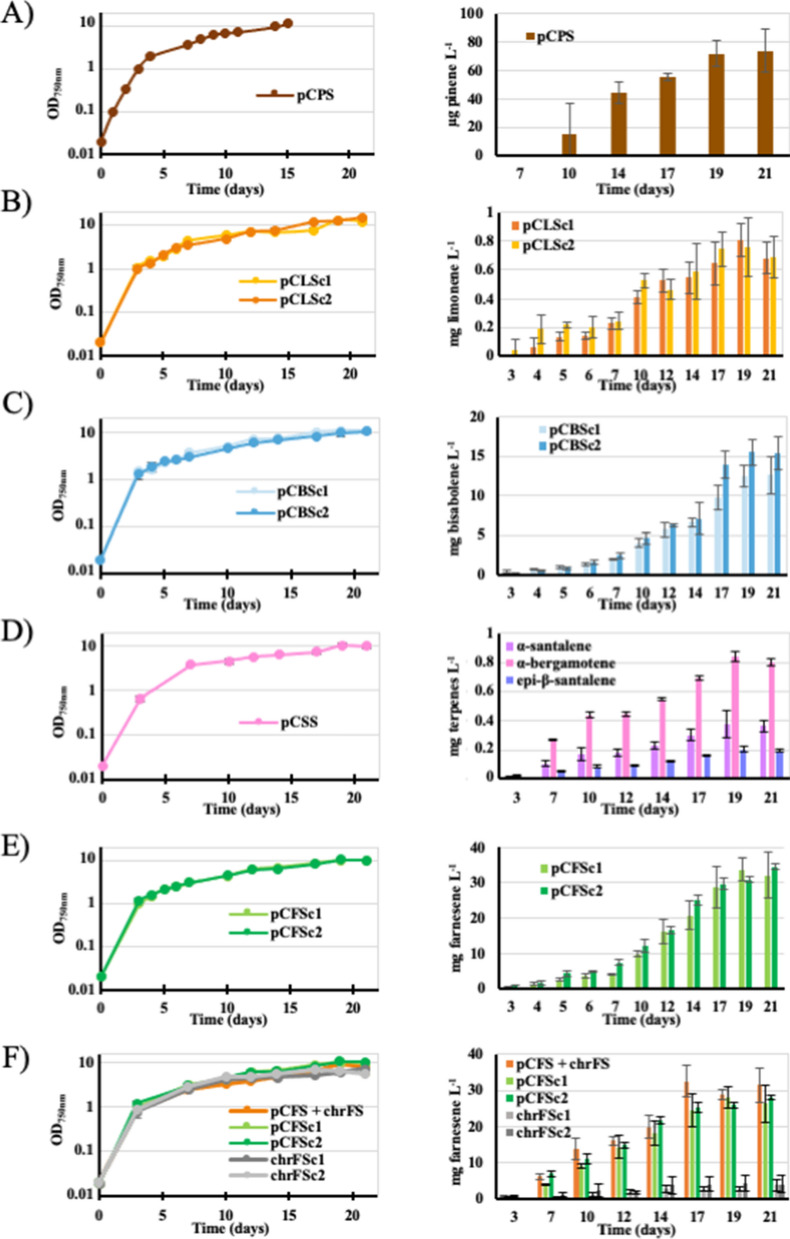
Simultaneous analysis of growth and terpene production of the *Synechocystis* PCC 6803 strains engineered in this study. Typical photoautotrophic growth (under a 20% (vol/vol) dodecane overlay) and terpene production of the *Synechocystis* strains expressing a terpene synthase gene from the following plasmids: **A** pCPS (pinene synthase), **B** pCLS (limonene synthase), **C** pCBS (bisabolene synthase), **D** pCSS (santalene synthase that produces α-santalene, α-*exo*-bergamotene and *epi*-β-santalene) and **E** pCFS (farnesene synthase). **F** Growth and farnesene production of the strains expressing the FS gene from either or both the pCFS plasmid and the slr0168 neutral chromosomal site (ChrFS). In most cases, two 2 clones (noted as c1, c2) were analyzed. Error bars represent standard deviation from *n* ≥ 2 biological replicates

**Fig. 4:**
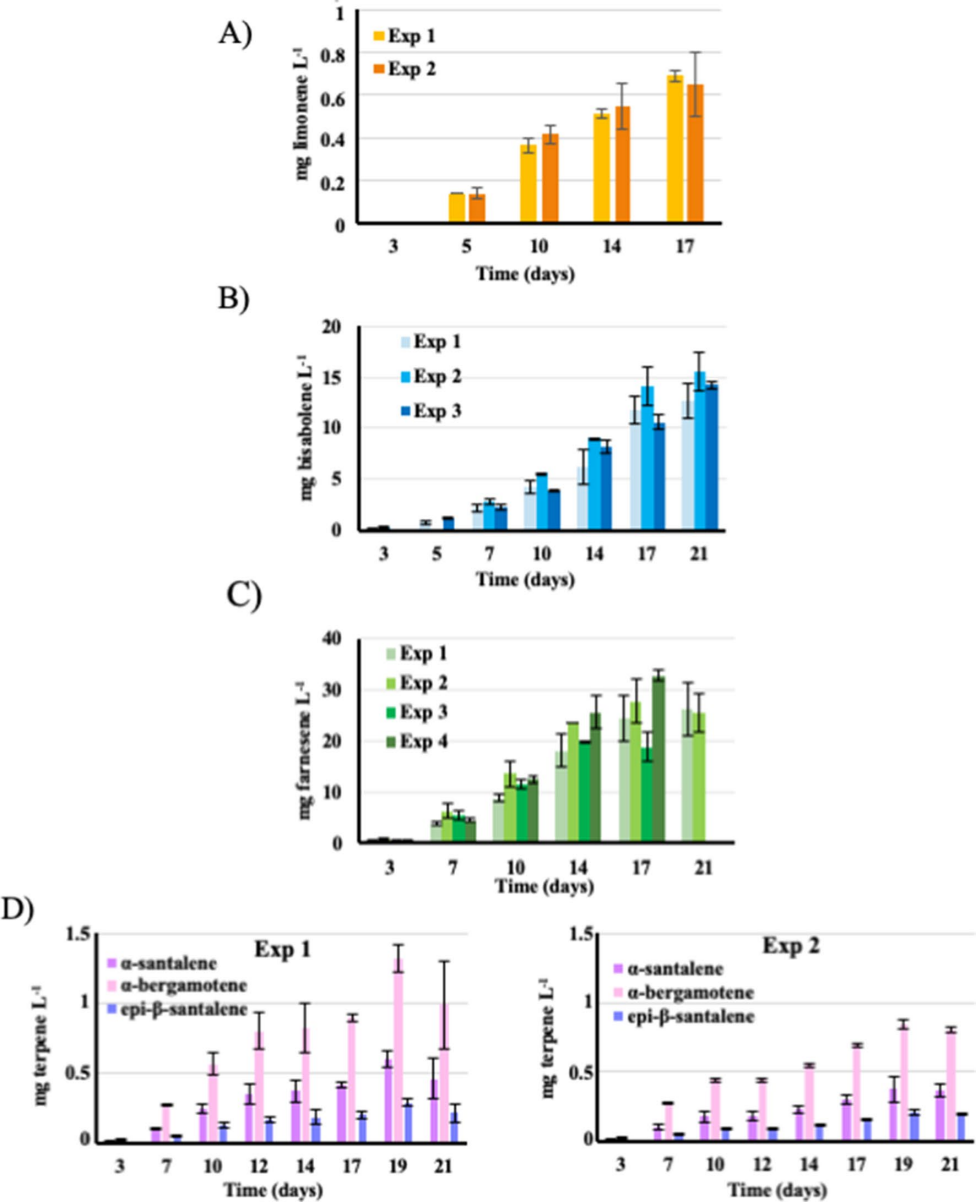
Influence of the duration of sub-cultivation of the engineered *Synechocystis* strains on terpene production. Cells sub-cultured under standard photoautotrophic conditions for variable periods of time (up to 5–9 months) were then grown under dodecane (20%, vol/vol) to assay terpene production during 17–21 days (Exp stands for experiment). **A** Limonene production by the pCLS strain sub-cultivated for various durations (up to 5 months) before testing. **B** Regular assays of α-bisabolene production by the pCBS strain sub-cultivated over a 5-month period of tests. **C** Regular tests of farnesene production by the pCFS strain sub-cultivated up to 9 months. **D** Regular tests of the production of α-santalene, α-*exo*-bergamotene and *epi*-β-santalene by the pCSS strain sub-cultivated up to 9 months. Error bars represent standard deviation from *n* ≥ 2 biological replicates

In this study we have engineered *Synechocystis* for the photosynthetic production of five chemically different high-value terpenes [2, 3, 6]: two monoterpenes (C_10_H_16_) limonene (cyclic molecule) and pinene (bicyclic), and three sesquiterpenes (C_15_H_24_) bisabolene (cyclic), farnesene (linear) and santalene (cyclic), using the same genetic strategy (Figs. 1, 2). Each codon-adapted terpene synthase genes were cloned behind the strong lambda-phage p*R* promoter of the RSF1010-derived pC expression vector (Additional file 1: Fig. S1) that replicates autonomously in *Synechocystis* at the same copy number (∼ 10 per cell) than the chromosome [8, 17]. All terpene producers appeared to grow well (Fig. 3 and Additional file 1: Fig. S2), and to be genetically stable (Fig. 4), an important finding rarely discussed in literature [2, 6], although it is known that recombinant cyanobacteria can be genetically unstable [18]. Our results are novel, because farnesene and santalene had not been produced before by *Synechocystis* or any cyanobacteria, respectively. They also show for the first time that *Synechocystis* can produce terpenes, when growing on urea instead of nitrate. Our results also indicate that *Synechocystis* is more prone to produce sesquiterpenes than monoterpenes (Additional file 1: Fig. S3K).

## Results and discussion

### Construction of the *Synechocystis* PCC 6803 strains expressing one of the five studied terpene synthase genes from the strong *lambda*-phage p*R* promoter of the RSF1010-derived pC plasmid vector

In this study, we tested and compared the ability of the best-studied (unicellular, robust) cyanobacterium *Synechocystis* to produce the five valuable terpenes [2], namely: bisabolene (C_15_H_24_), farnesene (C_15_H_24_), limonene (C_10_H_16_), pinene (C_10_H_16_) and the very-high priced [3] santalene (C_15_H_24_). For this purpose, we chose the following terpene synthases-encoding genes that worked well in cyanobacteria and/or other organisms [2, 3], namely: (i) the *Mentha spicata* 4S-limonene synthase gene (*ls,* [19]), (ii) the *Pinus taeda* ( +)-α-pinene synthase gene (*ps*, Additional file 1: Fig. S1A, B), (iii) the *Santalum album* santalene synthase (*ss*, Additional file 1: Fig. S1C, D), (iv) the *Abies grandis* E-α-bisabolene synthase (*bs*, Additional file 1: Fig. S1E, F) and (v) the *Picea abies* α-farnesene synthase (*fs*, Additional file 1: Fig. S1G, H). As done by previous workers [2] all terpenes synthase genes (*ts*) were adapted to the *Synechocystis* codon usage, and the chloroplast targeting sequence of the *ls* and *ps* genes were removed (deletion of the first 168 bp and 42 bp of *ls* of *ps*, respectively). Each codon-adapted *ts* gene synthesized by Eurofins Genomics or Twist Bioscience was flanked by a *Nde*I restriction site (CATATG) embedding its ATG start codon, and an *Eco*RI site downstream of its TAA stop codon (Additional file 1: Table S1 and Fig. S1). These enzymes were used to clone each *ts* coding sequence downstream of the strong λ*p*_*R*_-promoter and associated λ*cro* ribosome-binding site [17] of the pC plasmid vector [8] opened with the same enzymes (Additional file 1: Fig. S1A–H). The resulting plasmids pCBS, pCFS, pCLS, pCPS, and pCSS were verified by PCR, using appropriate oligonucleotide primers (Additional file 1: Table S1), and DNA sequencing (Mix2Seq Kit, Eurofins Genomics). They were then transferred by conjugation to *Synechocystis* [17, 19], yielding the strains represented in Fig. 2. In each case, two independent Sm^R^/Sp^R^ clones were selected and analyzed by PCR and DNA sequencing (Additional file 1: Table S1 and Fig. S1I) to verify that pCBS, pCFS, pCLS, pCPS, and pCSS plasmids replicate stably in *Synechocystis* without altering its photoautotrophic growth (regular verification were performed during 5–9 months (Figs. 3, 4). This finding is consistent with previous observations of other *Synechocystis* recombinant strains producing bisabolene [13–15] and limonene [11].

### The *Synechocystis* PCC 6803 strains harboring the pCPS plasmid produces pinene inefficiently

The production of pinene during the photoautotrophic growth of the *Synechocystis* strains propagating the pCPS plasmid, or the empty pC vector, was measured over a 21-day period of cultures (Fig. 3A). GC–MS analysis of dodecane overlay samples from the pCPS propagating strain, but not the pC negative-control strain, showed a peak with a similar retention time (Additional file 1: Table S2) and ion chromatogram than a pure standard of α-pinene (Additional file 1: Fig. S3A, B). The level of pinene production by the pCPS strain was low (∼80 μg L^−1^ after 21 days), in agreement to what was reported (∼40 μg L^−1^ in 168 h) by previous workers [12] who cloned into the slr2031 neutral chromosomal site of *Synechocystis* a similar codon-adapted *Pinus taeda* pinene synthase gene expressed from the strong *trc* promoter. Consequently, the pCPS engineered strain was not further studied.

### The *Synechocystis* PCC 6803 strains harboring the pCLS plasmids produces limonene weakly but stably

The production of limonene of the *Synechocystis* strains propagating pCLS, or the empty pC plasmid, during 21-day periods of photoautotrophic growth was analyzed by GC–MS (Fig. 3). The dodecane overlay samples from the pCLS strain, not the pC negative-control strain, showed a peak similar to that of a pure standard of S-(-)-limonene (Additional file 1: Table S2, Fig. S3C). The level of limonene production (∼0.25 mg L^−1^ after 7 days, and ∼0.7 mg L^−1^ after 21 days) (Fig. 3B) was similar to what was previously reported (∼0.25 mg L^−1^ after 7 days, no measurement after 7 days) after cloning into the slr2031 neutral chromosomal site a codon-adapted *Schizonepeta tenuifolia* limonene synthase gene expressed from the strong *trc* promoter [10]. Another group [11] observed higher levels of limonene production after cloning into the endogenous pCC5.2 plasmid a *trc*-expressed limonene synthase gene from either *Citrus limon* (∼1.0 mg L^−1^ after 7 days, no later measurement) or *Mentha spicata* (∼3.0 mg L^−1^ after 7 days, no later assay).

Unlike previous workers, we took care to verify that our pCLS limonene-producing strain is genetically stable (Fig. 4A). Indeed, no change in limonene production levels was observed during the regular tests performed over a 5-month period of sub-cultivation of the pCLS strain under photoautrophic conditions. This finding, rarely discussed in literature [2, 6], is important, because cyanobacteria engineered for chemical production can be genetically unstable [18], and stability of production is an important biotechnological criterion.

### The recombinant strain propagating the pCBS plasmid produces bisabolene efficiently and stably

Bisabolene production of the *Synechocystis* strains harboring the pCBS plasmid, or the empty pC vector, was measured over 21-day periods of photoautotrophic cultures (Fig. 3D). GC–MS analysis of the dodecane overlay samples from the pCBS strain, not the pC control, showed a peak typical to E-α-bisabolene (Additional file 1: Table S2, Fig. S3E, F). The level of α-bisabolene production (∼4–8 mg L^−1^ after 10–14 days; ∼14 mg L^−1^ after 21 days, Fig. 3) was similar to what was observed (∼5 mg L^−1^ after 12 days, no later measurement) after cloning a codon-optimized *Abies grandis* bisabolene synthase gene expressed from the strong *trc* promoter in an RSF1010-derived (pEEC1) plasmid [15]. Those production levels were also similar to what was reported by another group (∼7–8 mg L^−1^ after 6 days) who cloned a similar codon-optimized *Abies grandis* bisabolene synthase gene under the control of an IPTG inducible strong promoter (Ptic2op) into the slr0168 chromosomal site of *Synechocystis* [14]. We also took care to verify that our bisabolene producing pCBS strain is genetically stable (tested during 5 months, Fig. 4B), a point not discussed in previous reports.

Very interestingly, our results indicate that *Synechocystis* PCC6803 produces α-bisabolene ∼14 mg L^−1^) better than limonene (∼0.7 mg L^−1^), unlike what was observed [16] for the distantly related species *Synechococcus* PCC 7002 that produces limonene (∼4 mg L^−1^) better than α-bisabolene (∼0.6 mg L^−1^). Future works are required to characterize the molecular reasons of such differences between these two model cyanobacteria. Hence, we plead in favor of futures studies of the (i) potential of various cyanobacteria to produce various terpenes, and (ii) the transcriptomic, proteomic and metabolomic changes triggered by terpene production. Such studies should be of great help for choosing a cyanobacterium suitable for the photoproduction of an expected terpene.

### First time santalene production by an engineered cyanobacterium: the *Synechocystis* PCC 6803 strain propagating the pCSS plasmid produces santalene efficiently and stably

Santalene production by the *Synechocystis* strains propagating the pCSS plasmid, or the empty pC vector, was measured over 21-day periods of photoautotrophic growth (Fig. 3D). GC–MS analysis of dodecane overlay samples from the pCSS strain, not pC, showed several peaks typical to santalene isomers (α-santalene, *epi*-β-santalene and α-*exo*-bergamotene) present in a commercial Sandalwood oil (Additional file 1: Table S2, Fig. S3C, D). The overall production of santalene isomers (∼1.4 mg L^−1^ after 21 days) was not affected by the 9-month period of sub-cultivation of the pCSS strain under photoautotrophic conditions, prior to santalene measurement (Fig. 4D). These finding showed, for the first time, that a cyanobacterium can stably produce santalene and its high-value isomers [3].

### First time farnesene production by *Synechocystis* PCC 6803: the recombinant strain propagating the pCFS plasmid produces farnesene very efficiently and stably

Farnesene production by the *Synechocystis* strains harboring the pCFS plasmid or the empty pC vector was measured over 21-day periods of photoautotrophic growth (Fig. 3E, F). GC–MS analysis of dodecane overlay samples from the pCFS strain, not pC, showed a peak typical to α-farnesene (Additional file 1: Table S2, Fig. S3E, F).

**Fig. 5:**
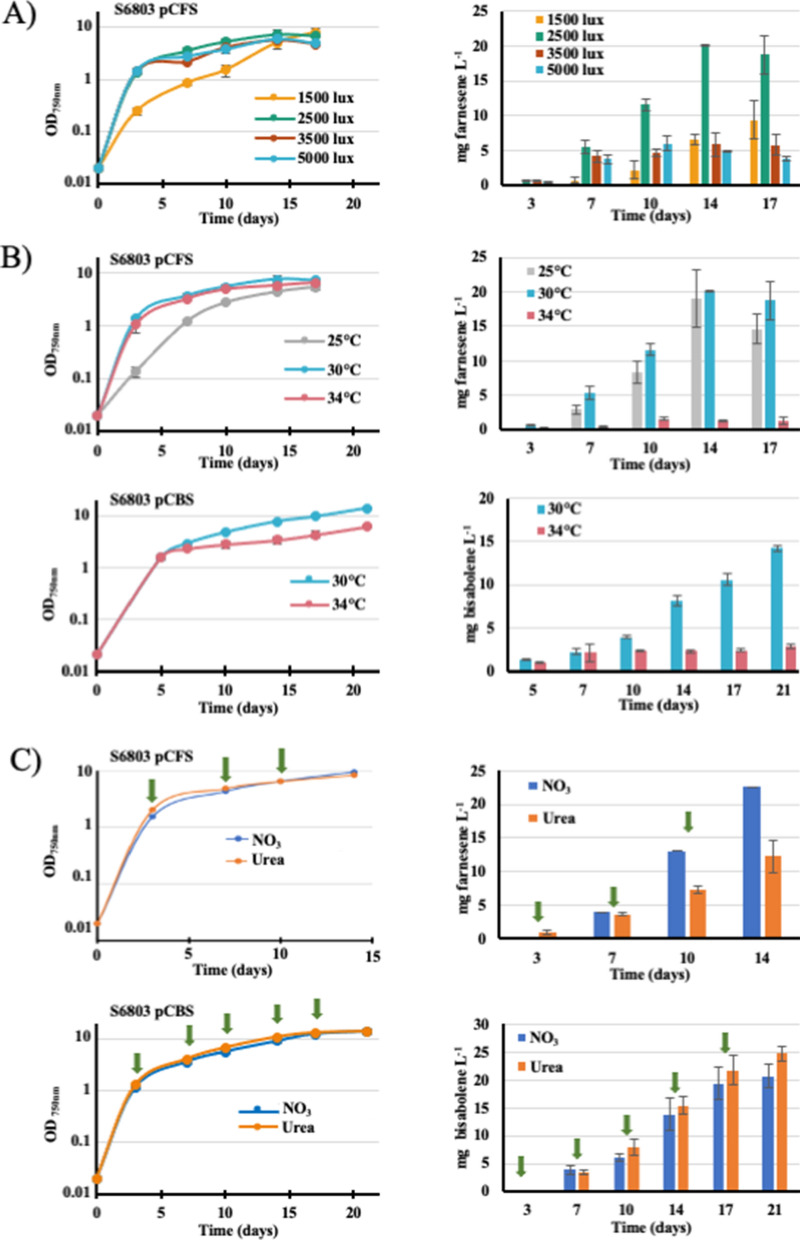
Influence of growth conditions on the production of farnesene and bisabolene by the *Synechocystis* strains engineered in this study. Cells pre-cultivated under standard photoautotrophic conditions were inoculated at the initial cell density of 10^6^ cells mL^−1^ (OD_750 nm_ = 0.02) and grown under dodecane (20% vol/vol) at the indicated lights (**A**) or temperatures (**B**), prior to measuring bisabolene and farnesene productions. **C** Cells washed and resuspended in nitrogen-free medium were overlaid with dodecane (20% vol/vol) after 3 days of incubation in growth medium supplemented at the indicated times (green arrows) with the same amounts of nitrogen provided as successive additions of either 4 mM nitrate (NO_3_) or 2 mM urea (CO(NH_2_)_2_). Error bars represent standard deviation from three biological replicates

Because light and temperature have strong effects on the photoautotrophic metabolism of cyanobacteria [4], we have studied their influence on the production of farnesene (Fig. 5). Both the standard light (2500 lx, 31.25 μE m^−2^ s^−1^) and temperature (30 °C) led to an active production (∼30 mg L^−1^ after 21 days, Figs. 4C and 5A, B). Interestingly, the level of farnesene production was decreased by a higher temperature (34 °C) or lights (3500 and 5000 lx) for as yet unknown reasons. The decrease observed at 34 °C suggests that the activity of the farnesene synthase could be sensitive to warm temperature. The (good) level of farnesene production observed in standard photoautotrophic conditions was unaffected by the duration of sub-cultivation of the pCFS strain (up to 6 months) prior to farnesene measurement (Fig. 4C). These findings showed, for the first time, that *Synechocystis* can produce farnesene efficiently and stably, i.e., two important biotechnological criteria [18].

The level of farnesene produced by *Synechocystis* (∼30 mg L^−1^ after 21 days) is higher than those reported for the phylogenetically distant cyanobacteria *Synechococcus* PCC 7942 (∼ 5 mg L^−1^ in 8 days; [20, 21] and *Anabaena* PCC 7120 (∼ 0.1 mg L^−1^ in 15 days; [22] expressing other farnesene synthase genes (from *Malus* domestica or *Norway spruce*, respectively) from different promoters propagated on different replicons. Furthermore, the genetic stability (or not) of these *Synechococcus* and *Anabaena* farnesene producers was not mentioned in these articles. Altogether, these differences in the nature of the cyanobacterial chassis and cloning (and expression) of the various farnesene genes employed make a rational comparison impossible. This situation emphasizes the great interest of truly comparative analyzes, where (i) one cyanobacterium is engineered the same way by the same workers for the photosynthetic production of different high-value terpenes as we presently report and/or (ii) several cyanobacteria are engineered the same way in the same laboratory for the production of one terpene. For these purposes the presently used shuttle vector based on the broad-host-range RSF1010 plasmid is advantageous (i) in being able to replicate autonomously in phylogenetically distant cyanobacteria and (ii) in allowing to clone various genes under the control of the same strong *pR* promoter [7, 8].

### First report that *Synechocystis* PCC 6803 can produce terpenes, using urea as the sole nitrogen source

As a step toward future economically feasible productions of terpenes coupled with wastewater treatment, we have tested the influence of urea, cheaper than nitrate and often present in natural waters [9], on the production of bisabolene and farnesene by the pCBS and pCFS recombinant strains growing under the efficient photoautotrophic conditions (30 °C; 2500 lx, 31.25 μE m^−2^ s^−1^) (Fig. 5A, B). In these assays, the same amount of nitrogen (4 mM) were supplied along cell growth as successive doses of 4 mM nitrate (NaNO_3_) or 2 mM urea (CO(NH_2_)_2_), because the growth of *Synechocystis* is impaired by higher concentration of urea (≥ 5 mM) supplied all at once [8], whereas it grows well on 17 mM in nitrate [23]. The results showed, for the first time, that *Synechocystis* can photosynthetically produce terpene using urea (cheaper than nitrate) as the sole nitrogen source (Fig. 5C). This finding is consistent with our recent report that the distantly related cyanobacterium *Cyanothece* PCC 7425 can produce limonene using either nitrate or urea as the nitrogen source [19].

### Comparison of farnesene production driven by the recombinant FS gene propagated by either or both the pCFS replicative plasmid and the slr0168 neutral chromosomal site

As the activity of an enzyme can be increased by increasing the copy number of the gene encoding it, we attempted to increase the farnesene production of the pCFS strain by cloning an extra copy of the p*R*-FS recombinant gene (FS expressed from the p*R* promoter) in the slr0168 neutral chromosomal site often used for gene cloning in *Synechocystis* [7]. Therefore, a p*R*-FS-Km^R^ DNA cassette was assembled (Additional file 1: Fig. S1J) and flanked by the two 300-bp DNA regions surrounding slr0168 to serve as platform for homologous recombination mediating targeted integration of the p*R*-FS-Km^R^ cartridge in slr0168. The resulting pTwist_NS-slr0168-FS plasmid (Additional file 1: Fig. S1J, K) was transformed to *Synechocystis* pCFS cells, where homologous DNA recombination integrated the p*R*-FS-Km^R^ cassette in the slr0168 site of all their chromosome copies (*Synechocystis* harbors 10 copies of its chromosome per cell [24]). The resulting strain expressing the p*R*-FS gene from both the pC plasmid (Additional file 1: Fig. S1G, H) and the chromosome (Additional file 1: Fig. S1L) was designated as pCFS + chrFS (Fig. 2).

In parallel, the pTwist_NS-slr0168-FS plasmid was transformed to *Synechocystis* wild-type cells, where the p*R*-FS-Km^R^ DNA cassette was integrated in the slr0168 site of all 10 chromosome copies (Additional file 1: Fig. S1J–L). The resulting strain named chrFS (Fig. 2) served to measure the level of farnesene production (Fig. 3) driven by only the chromosomal p*R*-FS gene (Additional file 1: Fig. S1J–L).

All three strains pCFS, chrFS and pCFS + chrFS grew equally well under standard photoautotrophic conditions, irrespectively of the presence of the terpene-trapping dodecane overlay (Additional file 1: Fig. S2). Farnesene was more efficiently produced (∼ sevenfold) by the pCFS strain (∼ 26–28 mg L^−1^ in 21 days, Fig. 3E) than by the chrFS strain (∼ 4 mg L^−1^ in 21 days, Fig. 3F), even though in both strains the FS gene was (i) expressed by the same (strong) p*R* promoter, and (ii) present at the same copy numbers (about 10 per cell) shared by the chromosome [24] and the pC plasmid [17]. This finding is consistent with a recent study showing that highest expression in *Synechocystis* can be obtained using RSF1010-derived replicative vectors as compared to chromosomal target sites [25].

More expectedly, the level of farnesene produced by the pCFS + chrFS strain (∼ 32 mg L^−1^ in 21 days) harboring the FS gene in both the pC plasmid and slr0168 chromosomal site was roughly equivalent to the sum of farnesene produced by the strains pFCS (∼ 26–28 mg L^−1^ in 21 days) and chrFS (∼ 4 mg L^−1^ in 21 days) (Fig. 3), which possess the FS gene in pC or slr0168, respectively.

The lower farnesene production of the chrFS strain as compared to the pCFS strain suggests that the p*R*-FS gene is less well expressed from the slr0168 chromosomal site than from the pC plasmid, thereby challenging the notion that slr0168 is a truly neutral cloning site, where various genes can be easily cloned with no negative influence on their expression. It is possible that the cloning of some genes in slr0168, which removes the DUF4114 domain of the Slr0168 protein that is conserved in other proteins [26], somehow interferes with the cloning and/or expression of the studied genes [27]. This hypothesis is supported by the fact that we could not clone the BS gene in slr0168. Other supposedly neutral cloning sites were recently reported to be refractory to gene cloning and/or expression [25, 28].

## Conclusions

In this work, we have performed the first comparative analysis of the ability of the best-studied cyanobacterium *Synechocystis* to photosynthetically produce chemically diverse terpenes of high value (bisabolene, farnesene, limonene, pinene and santalene). We showed for the first time that *Synechocystis* can stably produce farnesene, as well as santalene that had never been produced by a cyanobacterium before. We also showed for the first time that similar levels of terpene were produced by cells growing on nitrate (the classical nitrogen source of cyanobacteria) or urea (cheaper than nitrate). In addition, interestingly, our results indicate that *Synechocystis* produces sesquiterpenes better than monoterpenes (Additional file 1: Fig. S3K), suggesting that IPP is not limiting terpene production.

## Methods

### Bacterial strains and growth conditions

*E. coli* strains used for gene manipulations (TOP10 and NEB10 beta) or conjugative transfer (CM404 [17]) of RSF1010-derived replicative plasmids to *Synechocystis* PCC 6803 (*Synechocystis*) were grown at 37 °C (TOP10 and NEB10 beta) or 30 °C (CM404) on LB medium containing appropriate antibiotics: kanamycin (Km) 50 μg mL^−1^, streptomycin (Sm) 25 μg mL^−1^ or spectinomycin (Sp) 75 μg mL^−1^.

*Synechocystis* (wild-type strain obtained from the Institut Pasteur in Paris) was routinely grown at 30 °C, in liquid mineral medium (MM), i.e., BG-11 [23] enriched with 3.78 mM Na_2_CO_3_, under continuous agitation (140 rpm, Infors rotary shaker) and cool white light (2500 lx; 31.25 μE m^−2^ s^−1^), unless stated otherwise. Growth was monitored by regular measurements of optical density at 750 nm (OD_750_). For some experiments nitrate, the standard nitrogen source of MM (NaNO_3_ 17 mM), was replaced by urea. Practically, nitrate-grown cells were washed twice and resuspended in nitrogen free media that were supplemented with either urea (and NiSO_4,_ 2.5 μM) or nitrate, as indicated. *Synechocystis* was also grown on MM solidified with 1% Bacto Agar (Difco). Transformation and conjugative transfer of pC-derived plasmids (Additional file 1: Table S1) to *Synechocystis* were performed as previously described [19, 24], using appropriate antibiotics for selections: Km 50 μg mL^−1^, Sp 5 μg mL^−1^ and Sm 5 μg mL^−1^.

### Terpene collection and quantification by gas chromatography–mass spectrometry

*Synechocystis* strains engineered for terpene production were photo-autotrophically grown in the presence of selective antibiotics, in 250 mL Erlenmeyers containing 50 mL cell suspensions (or 125 mL Erlenmeyers containing 25 mL cultures for Fig. 5) overlaid with 20% (vol/vol) dodecane (analytical grade, Sigma-Aldrich) to trap terpenes. At time intervals, 300 μL aliquots of the dodecane overlay were collected. 1 μL of these dodecane samples were injected in a split mode 10:1 (S-(-)-limonene) or 5:1 (E-α-bisabolene and α-farnesene, α-pinene and santalene isomers) into a GC–MS apparatus (Trace1300 (GC) + ISQ LT (MS), ThermoScientific) equipped with a TG-5MS column (30 m × 0.25 mm × 0.25 µm) and operated with He carrier gas at 1.0 mL.min^−1^; ionization voltage 70 eV, transfer line temperature 250 °C; ion source temperature 200 °C. Analyses were carried out in the selected ion monitoring mode: m/z = 50–650, as we previously described [19]. The conditions used for the detection and quantification of all five studied terpenes (oven temperature programs, retention times, ion chromatograms identification, nature of the internal and calibration standards) are described in Additional file 1: Table S2 and Fig. S3. As expected, the C12 chain length of dodecane (C_12_H_26_) allowed easy chromatographic separation from limonene and pinene (C_10_H_16_) and bisabolene, farnesene and santalene (C_15_H_24_).

## Supplementary Information

**Additional file 1.** Additional tables and figures.

## Data Availability

The data sets generated during this study are included in this published article and its additional files.
