# Supplementary material for: Exploring the potential of the model cyanobacterium Synechocystis PCC 6803 for the photosynthetic production of various high-value terpenes
Source: Biotechnol Biofuels Bioprod. 2022 Oct 14;15:110. doi: 10.1186/s13068-022-02211-0 (PMC9564069; doi:10.1186/s13068-022-02211-0)

Figure S1 Construction of plasmids for high-level expression of terpene synthase genes in *Synechocystis*.

Fig. S1A. Construction of the pCPS plasmid for strong expression of the *Pinus taeda* α-pinene synthase gene adapted to the *Synechocystis* codon usage. All genes are represented by colored arrows pointing into the direction of their transcription. The **α-pinene synthase gene** of the pEX-A2-PS plasmid was cloned as a *Nde*I-*Eco*RI restriction fragment in the ($Sp^R/Sm^R, Cm^R$) pC vector (Table S1) opened with the same enzymes. The resulting ($Sp^R/Sm^R, Cm^S$) pCPS plasmid expresses the **α-pinene synthase gene** from the strong λ phage *pR* promoter (**PR**). The **Sp^R/Sm^R gene** is flanked by **transcription terminators** (**TT**) preventing readthrough of gene expression.

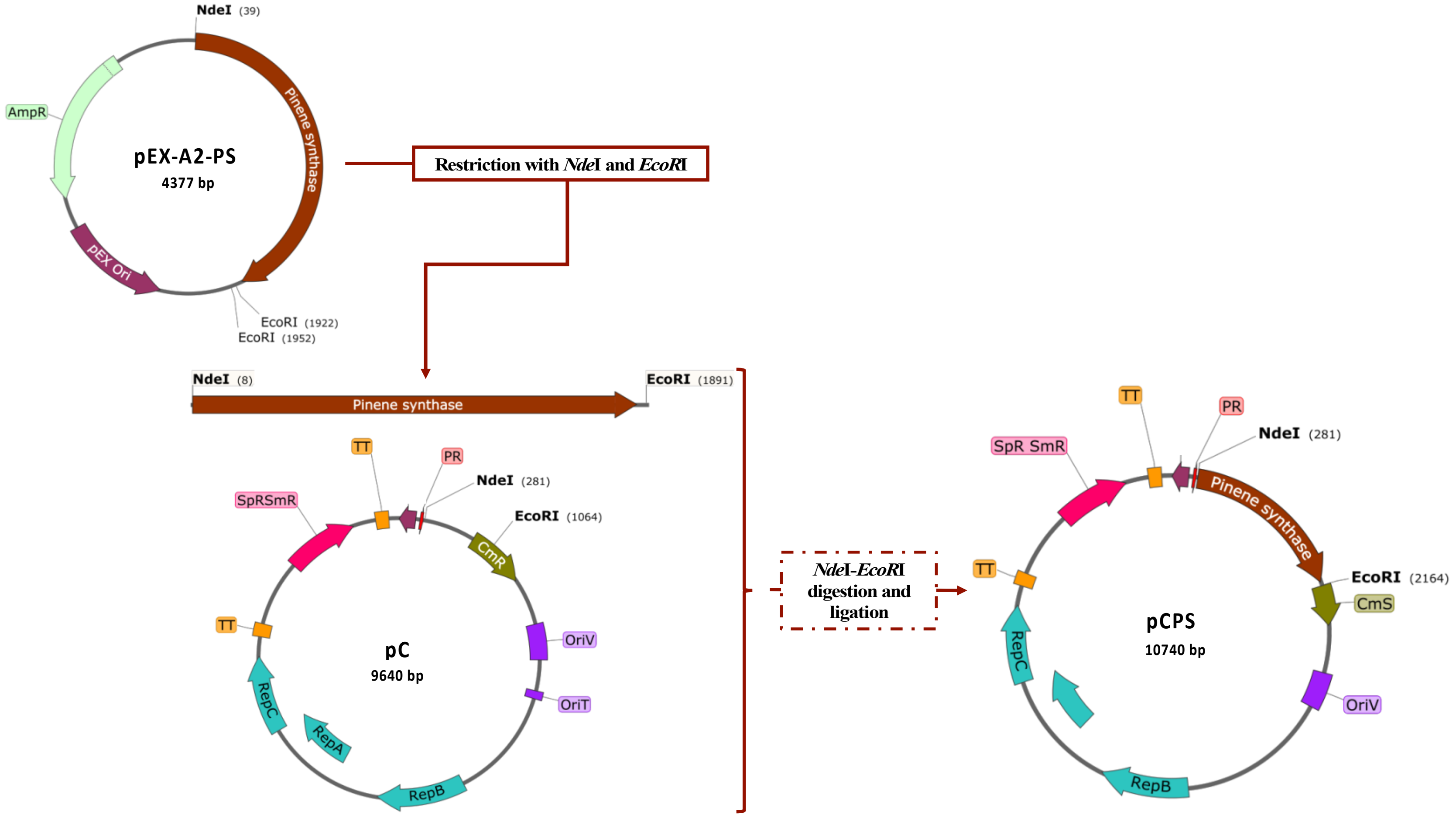

Fig. S1B. Relevant part of the nucleotide sequence of the pCPS plasmid. The *Nde*I (**catATG**) and *EcoR*I (**gaattc**) restriction sites were used for cloning the ***Pinus taeda* α-pinene synthase gene** downstream of the strong lambda-phage *pR* promoter (-35 box (**TTGACT**), -10 box (**GATAAT**) & transcription start site (**A**)) and associated ribosome binding site (**AAGGAGG**) of the pC vector.

5'-
GGCGACGTGCGTCCTCAAGCtgctcttgtgttaatggtttcttttttgtgctcatacgtta**aatctatcaccgcaagggataaatatctaacaccgtgcgtgTTGACTattttacc**
tctggcggtGATAATggttgcAtgtactaaggaggtcatATGCGCAGGACACTCTTCGGCTTTTCCCATGAACTTAAAGCTATTCACAGCACTGTACCTAACCTAGGTATGTGTAG
AGGAGGGAAGTCTATCGCCCCTAGCATGAGTATGTCTTCAACCACTTCGGTCAGCAATGAAGATGGGGTGCCTCGTCGAATTGCTGGTCACCATTCCAACTTGTGGGATGATGACT
CGATCGCCAGTTTGTCCACCTCATACGAAGCTCCGAGTTACCGTAAGAGGGCTGACAAGTTAATCGGAGAAGTAAAAAATATCTTTGATCTAATGAGCGTGGAAGATGGTGTGTTT
ACTAGCCCCCTATCCGATCTGCATCATCGACTTTGGATGGTGGATTCAGTGGAACGACTCGGCATCGATAGGCATTTTAAAGATGAGATCAATTCGGCGCTTGACCACGTGTACTC
TTACTGGACGGAGAAGGGAATAGGTCGGGGACGAGAGTCCGGAGTGACCGATTTAAATTCCACTGCACTCGGACTCCGCACGCTCCGGCTTCATGGCTATACGGTAAGTAGTCACG
TACTGGACCATTTTAAGAACGAAAAGGGCCAATTTACATGCTCAGCCATCCAGACTGAAGGGGAAATAAGAGATGTGCTAAACTTATTTAGAGCCTCTCTTATCGCCTTCCCAGGG
GAAAAGATCATGGAAGCTGCCGAAATATTTTCGACGATGTATCTCAAGGACGCGCTCCAAAAGATCCCACCCAGTGGTTTGAGCCAAGAAATCGAATACCTGCTTGAATTTGGGTG
GCACACAAACTTGCCCCGCATGGAAACACGGATGTATATTGATGTATTTGGGGAAGACACCACTTTTGAAACTCCCTATCTAATCCGCGAGAAGTTGCTAGAATTGGCCAAATTAG
AGTTCAATATCTTTCACAGCCTGGTGAAACGGGAATTGCAGTCTCTGTCTCGTTGGTGGAAAGACTACGGCTTTCCTGAGATTACCTTCAGCAGACATCGGCATGTGGAATATTAC
ACTTTAGCCGCTTGTATTGCAAATGATCCTAAACATAGCGCGTTTCGTTTGGGGTTCGGTAAAATTTCCCACATGATTACGATTCTGGATGATATTTATGATACCTTTGGGACTAT
GGAGGAGTTGAAATTACTGACTGCCGCCTTTAAACGGTGGGATCCCAGTTCAATTGAGTGTCTGCCGGACTATATGAAAGGTGTTTATATGGCTGTCTATGATAATATTAACGAAA
TGGCCCGGGAAGCACAAAAAATTCAGGGGTGGGATACCGTTTCTTACGCACGGAAATCCTGGGAAGCGTTCATTGGCGCGTATATTCAAGAGGCGAAATGGATTTCTTCCGGTTAT
CTGCCCACCTTTGACGAGTACTTGGAAAATGGTAAAGTTTCCTTTGGTAGTCGTATTACGACCTTGGAACCAATGCTGACCTTGGGCTTCCCCTTACCGCCACGTATTTTGCAAGA
AATTGATTTTCCGAGTAAATTTAATGATCTCATTTGCGCCATTTTGCGGTTAAAAGGCGATACCCAATGCTATAAAGCAGACCGCGCCCGCGGCGAGGAAGCCAGTGCTGTCAGTT
GTTATATGAAAGATCATCCCGGCATAACCGAAGAAGATGCGGTTAATCAGGTGAATGCTATGGTCGATAATTTAACCAAAGAATTAAATTGGGAATTATTACGGCCCGACTCCGGC
GTTCCAATTTCCTACAAAAAAGTGGCCTTTGATATTTGTCGCGTGTTTCACTATGGCTACAAATACCGCGATGGCTTCTCCGTGGCCTCCATTGAAATTAAAAATTTAGTTACCCG
CACCGTTGTTGAGACCGTTCCCTTATAAggatccatggtcgcgatcggtcgacgctagc**gaattccgtatggcaatgaaagacggtgagctggtgatatgggatagtgttcaccct**
tgttacac -3'

Amino acid sequence of the *Pinus taeda* α-pinene synthase (GenBank : Q84KL3)

MRRTLFGFSHELKAIHSTVPNLGMCRGGKSIAPSMSMSSTTSVSNEDGVPRRIAGHHSNLWDDDSIASLSTSYEAPSYRKRADKLIGEVKNIFDLMSVEDGVFTSPLSDLHHRLWM
VDSVERLGIDRHFKDEINSALDHVYSYWTEKGIGRGRESGVTDLNSTALGLRTLRLHGYTVSSHVLDHFKNEKGQFTCSAIQTEGEIRDVLNLFRASLIAFPGEKIMEAAEIFSTM
YLKDALQKIPPSGLSQEIEYLLEFGWHTNLPRMETRMYIDVFGEDTTFETPYLIREKLLELAKLEFNIFHSLVKRELQSLSRWWKDYGFPEITFSRHRHVEYYTLAACIANDPKHS
AFRLGFGKISHMITILDDIYDTFGTMEELKLLTAAFKRWDPSSIECLPDYMKGVYMAVYDNINEMAREAQKIQGWDTVSYARKSWEAFIGAYIQEAKWISSGYLPTFDEYLENGKV
SFGSRITTLEPMLTLGFPLPPRILQEIDFPSKFNDLICAILRLKGDTQCYKADRARGEEASAVSCYMKDHPGITEEDAVNQVNAMVDNLTKELNWELLRPDSGVPISYKKVAFDIC
RVFHYGYKYRDGFSVASIEIKNLVTRTVVETVPL*

Fig. S1C. Construction of the pCSS plasmid for strong expression of the *Santalum album santalene synthase* gene adapted to the *Synechocystis* codon usage. The ***santalene synthase* gene** of the pEX-A258-SS plasmid (Table S1) was cloned as a *Nde*I-*Eco*RI restriction fragment in the pC plasmid, yielding pCSS that expresses the ***santalene synthase* gene** from the strong *pR* promoter (**PR box**). **TT** indicates the **transcription terminators** flanking the ***Sp^R/Sm^R* marker**.

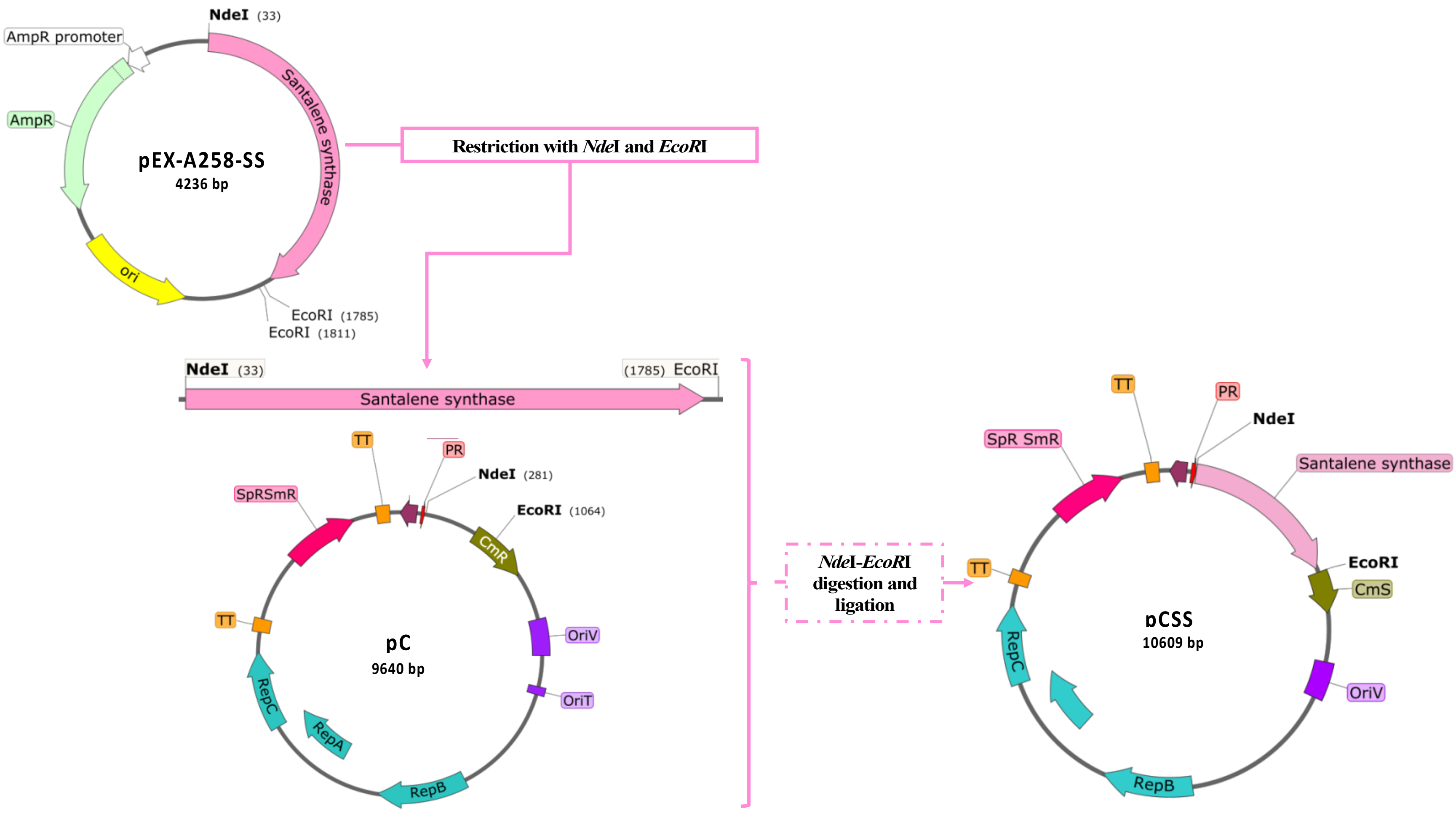

Fig.S1D. Relevant part of the nucleotide sequence of the pCSS plasmid. The *Nde*I (**cat**ATG) and *Eco*RI (**gaattc**) restriction sites were used for cloning the ***Santalum album* santalene synthase gene** downstream of the strong *pR* promoter (**TTGACT**, **GATAAT** & **A**) and associated ribosome binding site (**AAGGAGG**) of the pC vector.

5'-
ggcgacgtgcgtcctcaagctgctcttgtgttaatggtttctttttgtgctcatacgttaaatctatcaccgcaagggataaatatctaacaccgtgcgtg**TTGACT**attttacc
tctggcggt**GATAAT**ggttgc**A**tgtact**aaggagg**t**cat**ATGGACAGTTCTACAGCAACCGCGATGACTGCACCCTTTATTGACCCGACCGATCACGTAAACCTCAAAACTGATAC
CGACGCTAGTGAGAATCGCAGAATGGGCAATTATAAGCCGTCAATTTGGAACTACGATTTTCTTCAGTCCTTGGCTACCCACCATAATATAGTTGAAGAAAGACATCTGAAGCTTG
CTGAGAAACTCAAAGGCCAAGTGAAATTTATGTTTGGTGCACCCATGGAACCCCTGGCTAAATTGGAGTTAGTGGATGTGGTACAACGCCTAGGGCTAAATCATTTATTTGAGACG
GAAATAAAAGAAGCCTTATTTTCAATTTACAAAGATGGGAGCAATGGCTGGTGGTTTGGACACCTACATGCTACTTCCCTGCGCTTTAGGCTCTTGCGCCAGTGTGGGCTATTTAT
CCCACAAGATGTCTTCAAGACATTTCAAAACAAGACAGGAGAGTTCGATATGAAGCTTTGTGATAACGTAAAAGGTCTGTTGTCTTTGTACGAGGCAAGTTACTTGGGGTGGAAAG
GTGAGAACATTTTGGACGAGGCGAAGGCGTTTACTACGAAATGCCTTAAGTCGGCATGGGAGAATATCTCTGAGAAATGGTTAGCCAAACGAGTTAAGCACGCGCTGGCGCTCCCG
CTACACTGGAGAGTCCCCCGTATAGAGGCTCGCTGGTTTATTGAGGCCTACGAGCAAGAAGCGAACATGAACCCGACATTGCTCAAATTGGCCAAACTGGACTTCAACATGGTACA
GAGTATCCATCAAAAGGAAATTGGAGAATTAGCCCGATGGTGGGTGACCACCGGTTTGGACAAACTAGCCTTTGCTCGTAATAACCTGCTGCAGAGCTACATGTGGTCATGTGCTA
TTGCCTCCGATCCTAAGTTTAAGTTAGCCCGTGAAACTATTGTAGAAATTGGCTCTGTGTTGACGGTGGTAGATGACGGCTACGACGTTTACGGGTCCATCGATGAATTAGATTTG
TATACTAGCAGTGTGGAACGTTGGAGCTGTGTCGAAATTGATAAATTACCTAATACCCTTAAATTGATCTTTATGAGTATGTTTAATAAAACCAATGAAGTGGGGTTACGGGTGCA
ACACGAACGTGGCTATAACAGTATTCCTACCTTCATCAAAGCTTGGGTGGAACAGTGTAAATCGTATCAAAAAGAAGCCCGGTGGTTTCATGGGGGTCACACCCCCCCCCTGGAAG
AATATAGCTTAAATGGCTTGGTCTCCATTGGTTTTCCCTTACTCCTTATCACCGGGTATGTTGCCATTGCCGAAAATGAAGCCGCCTTGGACAAAGTCCATCCGCTACCCGACCTC
CTGCATTATTCTTCCCTTTTATCCCGGTTAATTAATGACATTGGAACCAGTCCAGATGAAATGGCCCGAGGTGATAACCTTAAATCCATCCATTGCTATATGAATGAAACCGGTGC
CTCTGAAGAAGTGGCCCGGGAACATATCAAGGGGGTTATTGAAGAAAATTGGAAAATTCTCAATCAGTGTTGCTTTGATCAGTCCCAATTCCAAGAACCCTTCATTACATTTAATT
TAAATAGTGTTCGGGGAAGCCATTTCTTTTATGAATTTGGTGATGGGTTCGGCGTGACTGACTCGTGGACGAAAGTGGATATGAAATCCGTGTTGATTGATCCAATTCCTTTAGGA
GAAGAATAAtcgcgactcgagctagcggatccgatgaacgcgttcagct**gaattc**cgtatggcaatgaaagacggtgagctggtgatatgggatagtgttcacccttgttacaccg
ttttccatgagcaaactgaaacgttttcatcgctctggagtgaataccacgacgatttccggcagtttctacacatatattcgcaagatgtggcgtgttacggtgaaaacctggcc
tatttccctaaagggtttattgagaatatgtttttcgtctcagccaatccctgggtgagtttcaccagttttgatttaaacgtggccaatatggacaacttcttcgcccccgtttt
caccatgggcaaat **-3'**

Amino acid sequence of the *Santalum album* santalene synthase (Genbank: ADO87000.1)

MDSSTATAMTAPFIDPTDHVNLKTDTDASENRRMGNYKPSIWNYDFLQSLATHHNIVEERHLKLAEKLKGQVKFMFGAPMEPLAKLELVDVVQRLGLNHLFETEIKEALFSIYKDG
SNGWWFGHLHATSLRFRLLRQCGLFIPQDVFKTFQNKTGEFDMKLCDNVKGLLSLYEASYLGWKGENILDEAKAFTTKCLKSAWENISEKWLAKRVKHALALPLHWRVPRIEARWF
IEAYEQEANMNPTLLKLAKLDFNMVQSIHQKEIGELARWWVTTGLDKLAFARNNLLQSYMWSCAIASDPKFKLARETIVEIGSVLTVVDDGYDVYGSIDELDLYTSSVERWSCVEI
DKLPNTLKLIFMSMFNKTNEVGLRVQHERGYNSIPTFIKAWVEQCKSYQKEARWFHGGHTPPLEEYSLNGLVSIGFPLLLITGYVAIAENEAALDKVHPLPDLLHYSSLLSRLIND
IGTSPDEMARGDNLKSIHCYMNETGASEEVAREHIKGVIEENWKILNQCCFDQSQFQEPFITFNLNSVRGSHFFYEFGDGFGVTDSWTKVDMKSVLIDPIPLGEE

Fig.S1E. Construction of the pCBS plasmid for strong expression of the *Abies grandis E-α-bisabolene synthase* gene adapted to the *Synechocystis* codon usage. All genes are represented by colored arrows. The ***E-α-bisabolene synthase* gene** of the pEX-A258-BS plasmid (Table S1) was cloned as a *Nde*I-*Eco*RI restriction fragment in the pC vector, yielding the pCBS plasmid that expresses the ***E-α-bisabolene synthase* gene** from the strong λ phage *pR* promoter (**PR box**). The **transcription terminators** flanking the ***Sp^R/Sm^R* gene** are indicated as **TT.**

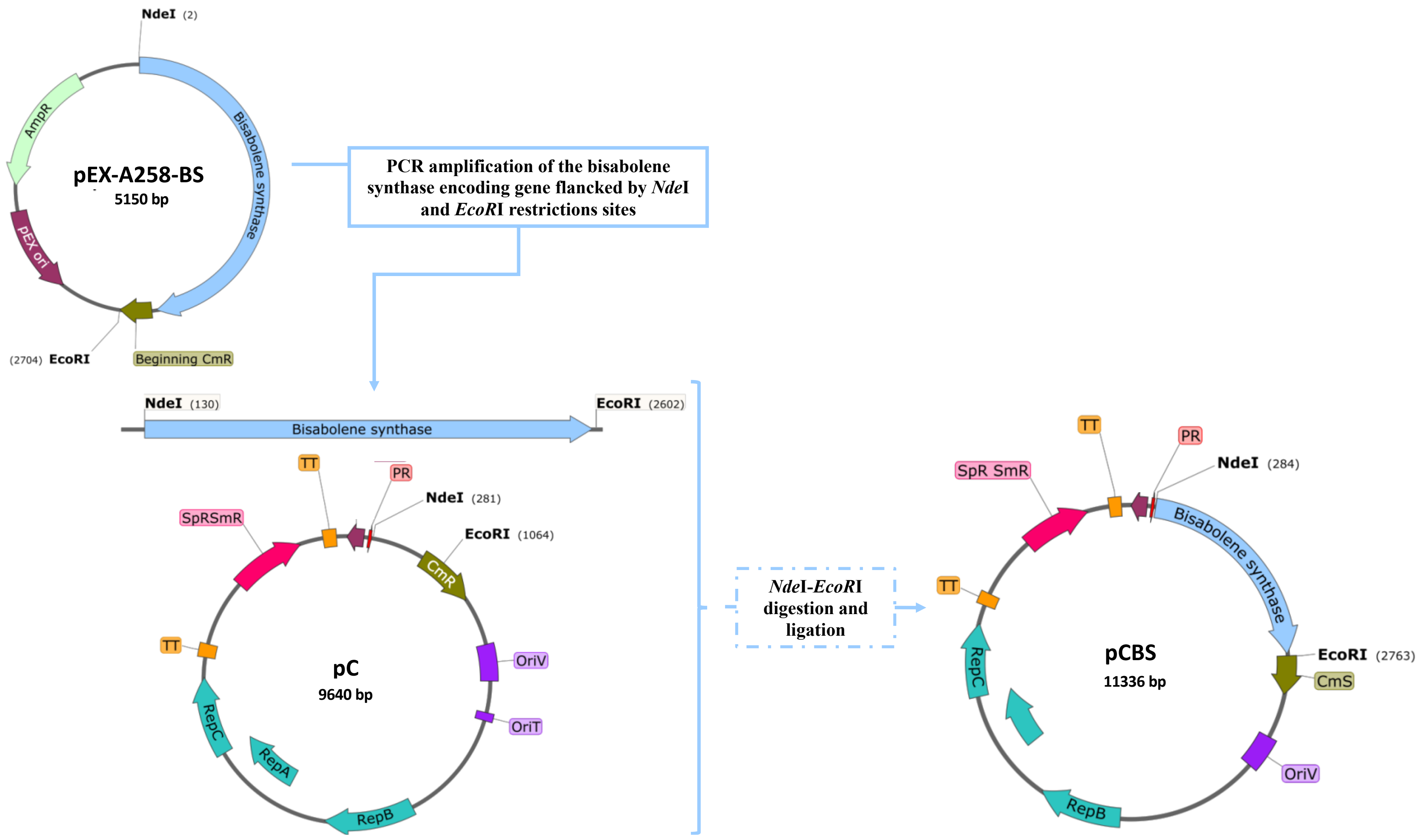

Fig. S1F. Relevant part of the nucleotide sequence of the pCBS plasmid. The *Nde*I (**cat**ATG) and *Eco*RI (**gaattc**) restriction site were used for cloning the ***E-α-bisabolene synthase* gene** downstream of the strong *pR* promoter (-35 box (**TTGACT**), -10 box (**GATAAT**) & transcription start site (**A**)) and associated ribosome binding site (**AAGGAGG**) of the pC vector.

5'-
GGCGACGTGCGTCCTCAAGCtgctcttgtgttaatggtttctttttttgtgctcatacgttaaatctatcaccgcaagggataaatatctaacaccgtgcgtgTTGACTattttacc
tctggcggtGATAATggttgcAtgtactaaggaggtcatATGGCAGGGGTCAGTGCTGTTTCCAAAGTCAGTAGTCTGGTTTGTGACCTGTCCTCTACGAGTGGTCTCATTCGACG
CACAGCTAATCCTCATCCGAACGTTTGGGGGTATGACCTAGTTCACTCGTTAAAAAGCCCTTACATAGACAGTAGTTACCGAGAGCGAGCGGAAGTCCTCGTTTCGGAAATCAAAG
CTATGCTGAACCCCGCTATAACGGGCGATGGCGAGTCCATGATTACTCCATCAGCCTATGACACCGCCTGGGTTGCTCGGGTTCCCGCTATTGATGGGTCAGCGCGGCCCCAATTT
CCACAAACTGTCGACTGGATACTCAAAAATCAGTTAAAGGACGGAAGTTGGGGCATACAATCTCATTTTTTACTCAGTGACCGACTCTTGGCTACACTCTCCTGCGTGCTCGTGCT
TCTCAAGTGGAACGTCGGCGATCTGCAAGTGGAACAAGGTATTGAGTTCATCAAGTCCAATTTGGAACTGGTCAAAGATGAGACCGACCAAGATAGCCTCGTGACCGATTTTGAGA
TCATCTTCCCCTCCCTACTACGTGAGGCACAGTCCCTAAGATTAGGTCTTCCGTACGACCTTCCCTACATCCACCTACTCCAAACTAAAAGGCAAGAACGCCTCGCAAAGTTGAGT
CGGGAGGAAATCTACGCCGTCCCAAGCCCATTACTCTACTCTTTGGAGGGCATCCAGGATATCGTCGAATGGGAGAGAATTATGGAGGTGCAATCCCAGGATGGCTCTTTTCTATC
TTCTCCTGCGTCGACTGCATGCGTCTTTATGCACACCGGCGACGCGAAGTGCCTTGAGTTCTTGAACTCTGTGATGATTAAGTTTGGTAATTTCGTACCCTGTTTGTACCCAGTAG
ATTTACTAGAGCGTTTACTTATTGTCGATAATATCGTTAGGTTAGGTATCTATAGGCACTTCGAGAAAGAAATCAAAGAAGCTCTGGACTATGTTTATAGGCATTGGAACGAGCGG
GGGATAGGCTGGGGGCGTCTCAACCCGATCGCGGACCTGGAAACTACAGCACTAGGTTTCCGCCTCCTTAGATTACATCGCTATAATGTGTCACCTGCAATCTTTGATAATTTCAA
GGATGCTAATGGTAAGTTCATTTGTAGCACAGGACAGTTCAATAAAGATGTAGCAAGTATGTTAAATTTATATAGAGCGAGCCAACTCGCGTTTCCTGGCGAGAACATTTTAGACG
AGGCTAAGTCCTTTGCTACGAAATACCTTAGAGAAGCTCTCGAGAAAAGTGAGACCAGCTCCGCTTGGAATAATAAGCAGAACCTTTCCCAAGAAATTAAGTACGCCCTTAAGACC
AGCTGGCATGCCTCGGTGCCGCGGGTAGAAGCCAAGCGCTATTGTCAAGTGTATCGTCCCGATTACGCGCGGATTGCTAAGTGTGTTTACAAGTTACCCTATGTTAACAATGAAAA
ATTTTTGGAATTAGGGAAATTGGACTTTAACATTATTCAAAGTATTCATCAAGAGGAGATGAAAAATGTCACCAGTTGGTTTCGGGACTCGGGACTACCCCTGTTTACCTTTGCGC
GGGAGCGTCCCCTGGAATTTTATTTTCTAGTTGCCGCGGGCACGTACGAACCACAGTACGCCAAATGTCGTTTTCTGTTTACCAAAGTAGCCTGTCTGCAGACCGTGCTAGATGAT
ATGTATGACACCTACGGTACCTTAGATGAATTGAAATTATTTACTGAAGCCGTTCGCCGCTGGGACTTGTCCTTTACTGAAAATTTGCCTGACTACATGAAATTGTGTTACCAGAT
TTATTATGATATTGTACATGAAGTGGCCTGGGAAGCCGAAAAAGAACAAGGGCGCGAACTGGTGAGTTTTTTTCGGAAAGGTTGGGAAGACTATTTATTGGGCTATTATGAAGAAG
CCGAATGGTTGGCCGCCGAATACGTGCCGACCTTGGATGAATATATTAAAAATGGTATTACGTCCATTGGGCAACGCATTCTTTTGCTATCGGGTGTGCTGATTATGGATGGCCAA
TTGTTGTCCCAAGAAGCCCTCGAAAAAGTGGATTATCCAGGACGGCGGGTGCTGACCGAACTGAATAGCCTGATTTCCCGTTTAGCCGATGATACTAAAACCTATAAAGCCGAAAA
GGCCCGCGGAGAACTGGCCTCTTCCATTGAATGCTACATGAAAGATCATCCCGAATGTACCGAAGAAGAAGCCCTGGATCACATTTATTCTATTTTGGAACCCGCCGTGAAAGAAT
TGACGCGTGAATTTTTAAAACCGGATGATGTGCCGTTTGCCTGCAAAAAAATGTTGTTTGAGGAAACCCGTGTGACCATGGTGATTTTTAAAGATGGAGATGGTTTTGGAGTGTCA
AAATTAGAAGTGAAAGATCATATTAAAGAATGCTTGATTGAACCGTTGCCACTATAAggatccatggtcgcgatcggtcgacgctagcgaattcCGTATGGCAATGAAAGACGGTG
AGCTGGTGATATGGGATAGTGTTCACCCTTGTTACAC-3'

Amino acid sequence of the *Abies grandis E-α-bisabolene synthase* (GenBank: AAK83562.1)

MAGVSAVSKVSSLVCDLSSTSGLIRRTANPHPNVWGYDLVHSLKSPYIDSSYRERAEVLVSEIKAMLNPAITGDGESMITPSAYDTAWVARVPAIDGSARPQFPQTVDWILKNQLK
DGSWGIQSHFLLSDRLLATLSCVLVLLKWNVGDLQVEQGIEFIKSNLELVKDETDQDSLVTDFEIIFPSLLREAQSLRLGLPYDLPYIHLLQTKRQERLAKLSREEIYAVPSPLLY
SLEGIQDIVEWERIMEVQSQDGSFLSSPASTACVFMHTGDAKCLEFLNSVMIKFGNFVPCLYPVDLLERLLIVDNIVRLGIYRHFEKEIKEALDYVYRHWNERGIGWGRLNPIADL
ETTALGFRLLRLHRYNVSPAIFDNFKDANGKFICSTGQFNKDVASMLNLYRASQLAFPGENILDEAKSFATKYLREALEKSETSSAWNNKQNLSQEIKYALKTSWHASVPRVEAKR
YCQVYRPDYARIAKCVYKLPYVNNEKFLELGKLDFNIIQSIHQEEMKNVTSWFRDSGLPLFTFARERPLEFYFLVAAGTYEPQYAKCRFLFTKVACLQTVLDDMYDTYGTLDELKL
FTEAVRRWDLSFTENLPDYMKLCYQIYYDIVHEVAWEAEKEQGRELVSFFRKGWEDYLLGYYEEAEWLAAEYVPTLDEYIKNGITSIGQRILLLSGVLIMDGQLLSQEALEKVDYP

Fig. S1G. Construction of the pCFS plasmid for strong expression of the *Picea abies α-farnesene synthase* gene adapted to the *Synechocystis* codon usage. The ***α-farnesene synthase* gene** of the pEX-A258-FS plasmid (Table S1) was cloned as a *Nde*I-*EcoR*I restriction fragment in the pC vector, generating the pCFS plasmid that expresses the ***α-farnesene synthase* gene** from the strong *pR* promoter (**PR box**). **TT** stands for the **transcription terminators** flanking the ***Sp^R/Sm^R* marker**.

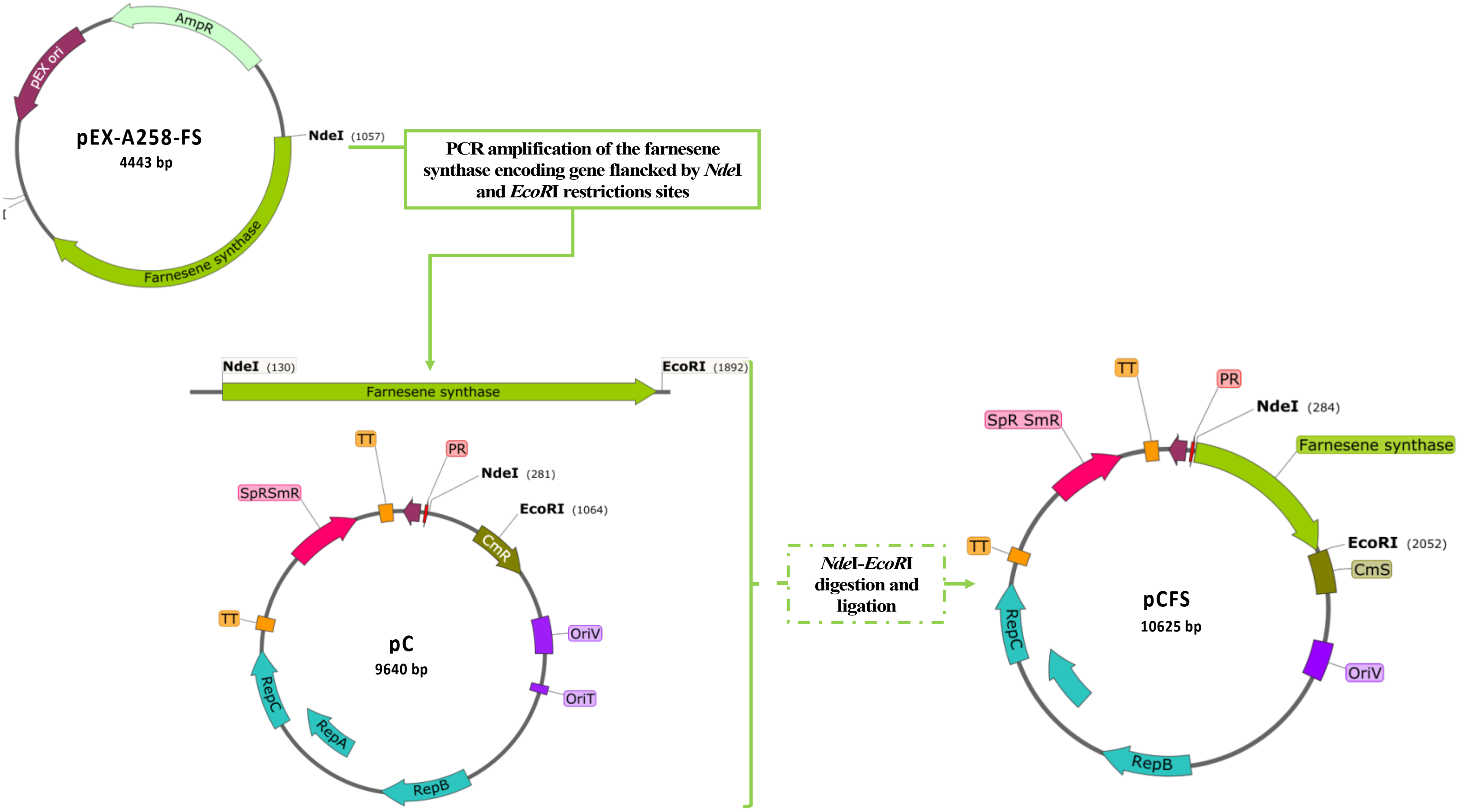

Fig. S1H. Relevant part of the nucleotide sequence of the pCFS plasmid. The *Nde*I (**cat**ATG) and *Eco*RI (**gaattc**) restriction sites were used for cloning the ***Picea abies α-farnesene synthase* gene** downstream of the strong *pR* promoter (**TTGACT**, **GATAAT** & **A**) and associated ribosome binding site (**AAGGAGG**) of the pC vector.

5'-
GGCGACGTGCGTCCTCAAGCtgctcttgtgttaatggtttctttttgtgctcatacgttaaatctatcaccgcaagggataaatatctaacaccgtgcgtgTTGACTattttacc
tctggcggtGATAATggttgcAtgtactaaggaggtcatATGGATTTGGCCGTTGAGATTGCCATGGACCTTGCAGTCGACGATGTTGAGAGAAGAGTAGGTGACTATCACTCGAA
TCTTTGGGACGATGATTTCATCCAATCGCTTTCCACACCCTACGGCGCGTCGTCCTATCGGGAGCGAGCAGAGCGCTTGGTCGGTGAAGTGAAGGAAATGTTTACCTCAATTAGTA
TTGAGGACGGGGAACTTACCTCAGACCTGTTGCAGCGCTTGTGGATGGTAGACAATGTGGAGCGCTTAGGGATTTCCAGACACTTTGAGAATGAGATCAAAGCTGCGATTGATTAT
GTGTACAGCTACTGGAGTGACAAAGGCATAGTCCGCGGTCGAGACTCCGCAGTTCCTGACTTAAATTCCATCGCCCTCGGTTTCCGTACATTACGTTTACACGGTTACACTGTGTC
AAGCGACGTTTTTAAGGTCTTTCAGGATCGAAAGGGAGAATTTGCATGTTCTGCTATTCCGACCGAGGGTGACATTAAAGGAGTGCTTAATTTGCTCCGCGCGAGTTACATCGCGT
TTCCCGGTGAAAAGGTAATGGAGAAAGCGCAAACCTTTGCTGCCACTTACTTGAAAGAGGCGTTGCAAAAGATTCAAGTGTCTTCTCTCTCCAGAGAGATTGAGTACGTGCTCGAA
TACGGGTGGCTAACCAACTTTCCGAGGTTAGAAGCGAGGAATTACATTGACGTTTTTGGGGAAGAAATCTGCCCTTATTTCAAGAAACCATGCATTATGGTGGATAAATTACTAGA
GCTGGCTAAGCTCGAATTTAACCTATTTCACTCTCTACAACAGACTGAACTAAAACATGTATCCAGGTGGTGGAAGGATAGTGGATTTTCCCAGCTCACGTTTACGCGGCATCGGC
ACGTGGAATTTTACACCTTGGCTAGCTGCATAGCCATTGAACCCAAACATTCCGCGTTCCGGCTCGGCTTTGCTAAAGTGTGTTATCTAGGGATAGTCCTAGATGATATTTATGAT
ACATTCGGAAAGATGAAGGAACTAGAACTGTTTACTGCGGCGATTAAGCGGTGGGATCCTTCTACTACGGAATGTTTACCCGAATACATGAAAGGTGTTTACATGGCTTTTTATAA
TTGTGTGAATGAACTTGCCTTACAAGCTGAAAAAACCCAAGGCCGTGATATGTTGAACTATGCTCGCAAAGCCTGGGAAGCCCTGTTTGATGCCTTTCTGGAAGAAGCCAAGTGGA
TTTCCAGCGGTTATCTGCCAACCTTTGAAGAATATCTCGAAAATGGCAAAGTGAGTTTCGGCTATCGTGCCGCCACCTTGCAGCCAATCCTAACCTTAGATATCCCTCTGCCCCTG
CATATTTTACAGCAGATTGATTTTCCCTCCCGCTTTAATGATTTGGCCAGTAGTATCTTGCGTCTGCGCGGGGATATTTGTGGCTATCAAGCCGAACGGAGCCGGGGCGAAGAAGC
AAGTTCCATTTCCTGTTATATGAAAGATAACCCGGGAAGCACTGAAGAAGATGCCTTGTCTCATATCAATGCCATGATTAGCGATAATATTAATGAATTGAACTGGGAATTATTGA
AACCCAACAGTAACGTGCCCATTAGTAGTAAAAAACATGCCTTTGATATTTTGCGTGCCTTTTATCATCTGTATAAATATCGGGATGGCTTTAGTATTGCCAAAATTGAAACGAAA
AACTTAGTAATGCGTACCGTACTGGAACCCGTTCCCATGTAAggatccatggtcgcgatcggtcgacgctagc**gaattc**CGTATGGCAATGAAAGACGGTGAGCTGGTGATATGGG
ATAGTGTTCACCCTTGTTACAC-3'

Amino acid sequence of the *Picea abies α-farnesene synthase*

MDLAVEIAMDLAVDDVERRVGDYHSNLWDDDFIQSLSTPYGASSYRERAERLVGEVKEMFTSISIEDGELTSDLLQRLWMVDNVERLGISRHFENEIKAAIDYVYSYWSDKGIVRG
RDSAVPDLNSIALGFRTLRLHGYTVSSDVFKVFQDRKGEFACSAIPTEGDIKGVLNLLRASYIAFPGEKVMEKAQTFAATYLKEALQKIQVSSLSREIEYVLEYGWLTNFPRLEAR
NYIDVFGEEICPYFKKPCIMVDKLLELAKLEFNLFHSLQQTELKHVSRWWKDSGFSQLTFTRHRHVEFYTLASCIAIEPKHSAFRLGFAKVCYLGIVLDDIYDTFGKMKELELFTA
AIKRWDPSTTECLPEYMKGVYMAFYNCVNELALQAEKTQGRDMLNYARKAWEALFDAFLEEAKWISSGYLPTFEEYLENGKVSFGYRAATLQPILTLDIPLPLHILQQIDFPSRFN
DLASSILRLRGDICGYQAERSRGEEASSISCYMKDNPGSTEEDALSHINAMISDNINELNWELLKPNSNVPISSKKHAFDILRAFYHLYKYRDGFSIAKIETKNLVMRTVLEPVPM
*

Figure S1I. PCR verification of the structure of the pC family of plasmids (pC, pCLS, pCBS, pCFS, pCPS, pCSS) expressing the studied terpene-synthase-encoding genes in *Synechocystis* PCC 6803.

a. Schematic representation of the relevant region of the pC (empty) plasmid vector and its derivative expressing the terpene synthase genes (large coloured arrows) from the strong lambda-phage pR promoter (small red trianges). PCR primers (pCF1_Fw and pCF1_Rv, additional file 1: Table S1B) and resulting DNA products are indicated by blue triangles and double arrows, respectively.

b. typical UV-light image of the corresponding agarose gels used to analyze two clones of each studied strain excepted for pCSS. M: DNA size marker = 1 kb Plus DNA Ladder Thermo Scientific GeneRuler. C^{-} correspond to a negative control (no pC derived plasmid).

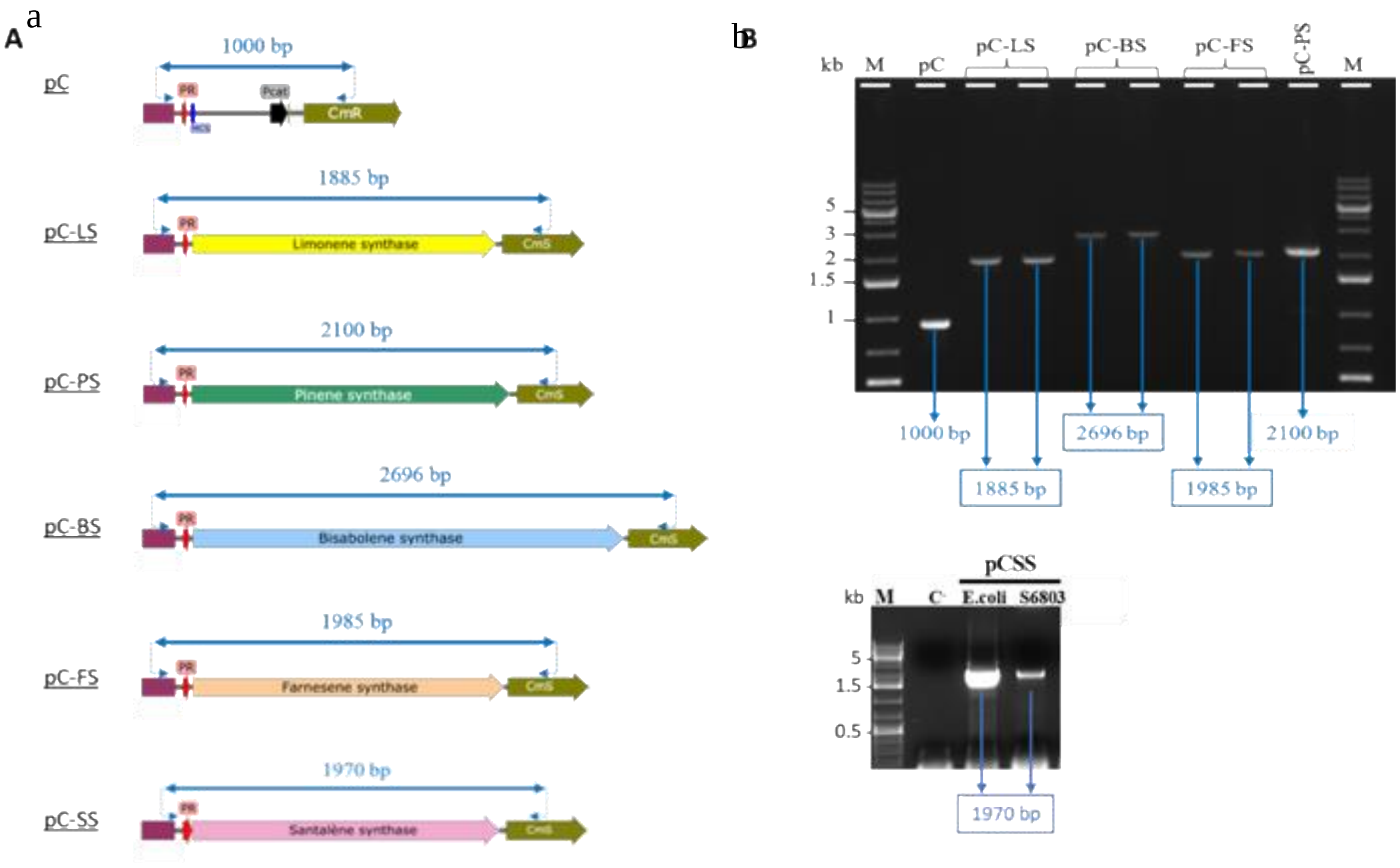

Figure S1J. Construction of the *Synechocystis* strain harboring the *Picea abies α-farnesene synthase* gene in the slr0168 chromosome site. First, the ***pR-FS*** gene (expression of the ***α-farnesene synthase* gene** from the ***pR*** promoter) and the **Km^{R}** marker were PCR amplified from the plasmids pCFS and pUC4K, respectively (Table S1). Second, the pTwist_NS-slr0168 plasmid was opened at its unique *Eco*RV restriction site flanked by the two 300 bp chromosomal DNA regions surrounding slr0168. Third, all three DNA cassettes (***pR-FS***, **Km^{R}** and pTwist_NS-slr0168) were assembled by Gibson® cloning. The resulting pTwist_NS-slr0168-FS plasmid was transformed to *Synechocystis* to insert the ***pR-FS*-Km^{R}** cassette in slr0168.

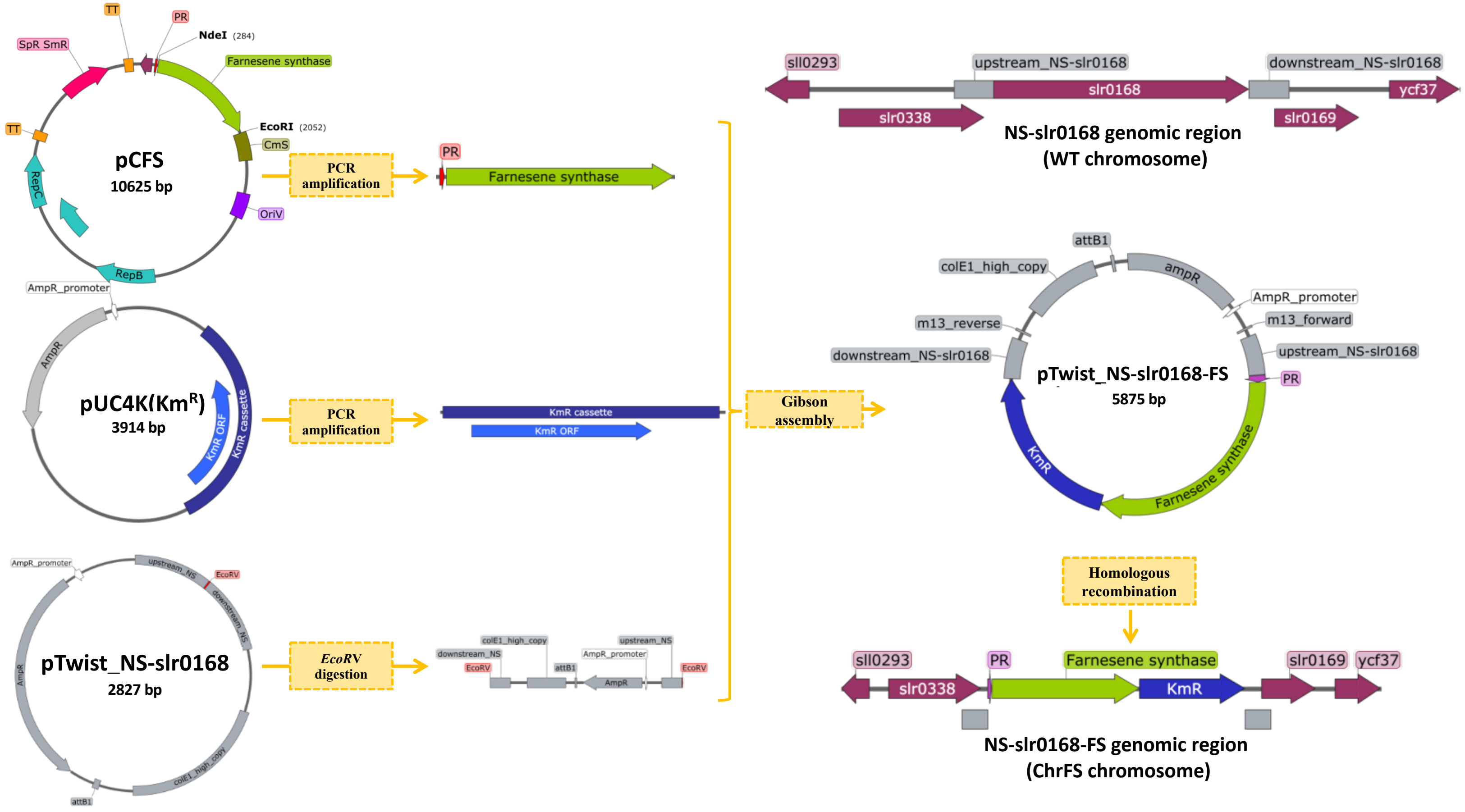

Fig. S1K. Nucleotide sequence of the *pR*-FS-Km^R DNA cassette cloned in slr0168 chromosome neutral site. The ***pR*-FS-Km^R** DNA cassette embedded in an *Eco*RV restriction site **GAT/ATC** flanked by DNA sequences **upstream** and d**ownstream** of slr0168, expressed the codon-adapted ***α-farnesene synthase* gene** from the strong *pR* promoter (**TTGACT**, **GATAAT** & **A**) and associated ribosome binding site (**AAGGAGG**).

5'-
AAAAGGTTTACACCAGTAATATTGAACCTCTCCACGCTGAATTAGAACATTTTATTCATTGTGTTAGGGGAGGTGATCAACCCTCAGTGGGGGGAGAACAGGCCCTCAAGGCCCTG
AAGTTAGCCAGTTTAATTGAAGAAATGGCCCTGGACAGTCAGGAATGGCATGGGGGGGAAGTTGTGACAGAATATCAAGATGCCACCCTGGCCCTCAGTGCGAGTGTTTAAATCAA
CTTAATTAATGCAATTATTGCGAGTTCAAACTCGATAACTTTGTGAAATATTACTGTTGAATTAATCTGATTTGACTattttacctctggcggtGATAATggttgcAtgtactaag
gaggtcatATGGATTTGGCCGTTGAGATTGCCATGGACCTTGCAGTCGACGATGTTGAGAGAAGAGTAGGTGACTATCACTCGAATCTTTGGGACGATGATTTCATCCAATCGCTT
TCCACACCCTACGGCGCGTCGTCCTATCGGGAGCGAGCAGAGCGCTTGGTCGGTGAAGTGAAGGAAATGTTTACCTCAATTAGTATTGAGGACGGGGAACTTACCTCAGACCTGTT
GCAGCGCTTGTGGATGGTAGACAATGTGGAGCGCTTAGGGATTTCCAGACACTTTGAGAATGAGATCAAAGCTGCGATTGATTATGTGTACAGCTACTGGAGTGACAAAGGCATAG
TCCGCGGTCGAGACTCCGCAGTTCCTGACTTAAATTCCATCGCCCTCGGTTTCCGTACATTACGTTTACACGGTTACACTGTGTCAAGCGACGTTTTTAAGGTCTTTCAGGATCGA
AAGGGAGAATTTGCATGTTCTGCTATTCCGACCGAGGGTGACATTAAAGGAGTGCTTAATTTGCTCCGCGCGAGTTACATCGCGTTTCCCGGTGAAAAGGTAATGGAGAAAGCGCA
AACCTTTGCTGCCACTTACTTGAAAGAGGCGTTGCAAAAGATTCAAGTGTCTTCTCTCTCCAGAGAGATTGAGTACGTGCTCGAATACGGGTGGCTAACCAACTTTCCGAGGTTAG
AAGCGAGGAATTACATTGACGTTTTTGGGGAAGAAATCTGCCCTTATTTCAAGAAACCATGCATTATGGTGGATAAATTACTAGAGCTGGCTAAGCTCGAATTTAACCTATTTCAC
TCTCTACAACAGACTGAACTAAAACATGTATCCAGGTGGTGGAAGGATAGTGGATTTTCCCAGCTCACGTTTACGCGGCATCGGCACGTGGAATTTTACACCTTGGCTAGCTGCAT
AGCCATTGAACCCAAACATTCCGCGTTCCGGCTCGGCTTTGCTAAAGTGTGTTATCTAGGGATAGTCCTAGATGATATTTATGATACATTCGGAAAGATGAAGGAACTAGAACTGT
TTACTGCGGCGATTAAGCGGTGGGATCCTTCTACTACGGAATGTTTACCCGAATACATGAAAGGTGTTTACATGGCTTTTTATAATTGTGTGAATGAACTTGCCTTACAAGCTGAA
AAAACCCAAGGCCGTGATATGTTGAACTATGCTCGCAAAGCCTGGGAAGCCCTGTTTGATGCCTTTCTGGAAGAAGCCAAGTGGATTTCCAGCGGTTATCTGCCAACCTTTGAAGA
ATATCTCGAAAATGGCAAAGTGAGTTTCGGCTATCGTGCCGCCACCTTGCAGCCAATCCTAACCTTAGATATCCCTCTGCCCCTGCATATTTTACAGCAGATTGATTTTCCCTCCC
GCTTTAATGATTTGGCCAGTAGTATCTTGCGTCTGCGCGGGGATATTTGTGGCTATCAAGCCGAACGGAGCCGGGGCGAAGAAGCAAGTTCCATTTCCTGTTATATGAAAGATAAC
CCGGGAAGCACTGAAGAAGATGCCTTGTCTCATATCAATGCCATGATTAGCGATAATATTAATGAATTGAACTGGGAATTATTGAAACCCAACAGTAACGTGCCCATTAGTAGTAA
AAAACATGCCTTTGATATTTTGCGTGCCTTTTATCATCTGTATAAATATCGGGATGGCTTTAGTATTGCCAAAATTGAAACGAAAAACTTAGTAATGCGTACCGTACTGGAACCCG
TTCCCATGTAAgacctgcagggggggggggggaaagccacgttgtgtctcaaaatctctgatgttacattgcacaagataaaaatatatcatcatgaacaataaaactgtctgctta
cataaacagtaatacaaggggtgttATGAGCCATATTCAACGGGAAACGTCTTGCTCGAGGCCGCGATTAAATTCCAACATGGATGCTGATTTATATGGGTATAAATGGGCTCGCG
ATAATGTCGGGCAATCAGGTGCGACAATCTATCGATTGTATGGGAAGCCCGATGCGCCAGAGTTGTTTCTGAAACATGGCAAAGGTAGCGTTGCCAATGATGTTACAGATGAGATG
GTCAGACTAAACTGGCTGACGGAATTTATGCCTCTTCCGACCATCAAGCATTTTATCCGTACTCCTGATGATGCATGGTTACTCACCACTGCGATCCCCGGGAAAACAGCATTCCA
GGTATTAGAAGAATATCCTGATTCAGGTGAAAATATTGTTGATGCGCTGGCAGTGTTCCTGCGCCGGTTGCATTCGATTCCTGTTTGTAATTGTCCTTTTAACAGCGATCGCGTAT
TTCGTCTCGCTCAGGCGCAATCACGAATGAATAACGGTTTGGTTGATGCGAGTGATTTTGATGACGAGCGTAATGGCTGGCCTGTTGAACAAGTCTGGAAAGAAATGCATAAGCTT
TTGCCATTCTCACCGGATTCAGTCGTCACTCATGGTGATTTCTCACTTGATAACCTTATTTTTGACGAGGGGAAATTAATAGGTTGTATTGATGTTGGACGAGTCGGAATCGCAGA
CCGATACCAGGATCTTGCCATCCTATGGAACTGCCTCGGTGAGTTTTCTCCTTCATTACAGAAACGGCTTTTTCAAAAATATGGTATTGATAATCCTGATATGAATAAATTGCAGT
TTCATTTGATGCTCGATGAGTTTTTCTAAtcagaattggttaattggttgtaacactggcagagcattacgctgacttgacgggacggcggctttgttgaataaatcgaacttttg
ctgagttgaaggatcagatcacgcatcttcccgacaacgcagaccgttccgtggcaaagcaaaagttcaaaatcaccaactggtccacctacaacaaagctctcatcaaccgtggc
tccctcactttctggctggatgatggggcgattcaggcctggtatgagtcagcaacaccttcttcacgaggcagacctcagcgcccccccccccctgcaggtcATCCTCAGGGGCA
TTATCGGAGCAAGCCGATATTCAGGTAAGATGGGGCGGTGCTTTTGTGGGTTGATCCATTAGCTTTAACCCCATTGTCTCCACTGACGGTAATAGTTTTCCCCACTGCGATTAATT
GTTTTGTTTGGAGGAGGGACTGCCCCACCGTCAGCCCGCCGCCATAGTTTTGCATTTCCACTGCCATGGGCATCGAACTCCGTAGTTACGTTTACCTGGATAGTCTCCAGTCCCAA
CATGCAGCCTACATTGGGACGGTGGCCTCCGGCTTTTTGCCGCTACCGGGGGATTGTT -3'

Fig. S1L. PCR verification showing that the *pR*-FS-Km^{R} DNA cassette expressing the *Picea abies farnesene synthase* gene is properly integrated in the slr0168 site of all chromosome copies of *Synechocystis* PCC 6803 strains harboring or not the pFCS plasmid.

Upper part: schematic representation of the genome of the *Synechocystis* strains expressing the *farnesene synthase* gene from the slr0168 chromosomal site alone (chrFS strain) or together with the pCFS plasmid (chrFS + pCFS strain). Cells are shown as green oval shapes representing their chromosome (orange line) arbitrarily showed as attached to the cell membrane to distinguish it from the pCFS plasmid. The strong λ phage *pR* promoter directing the expression of the FS gene is shown as the red triangle.

Lower part: typical UV-light image of the agarose gels showing the 3584 bp PCR product (amplified with the NSslr0168_a_Fwd and NSslr0168_b_rev primers, additional file 1: Table S1B) corresponding to the *pR*-FS-Km^{R} chromosomal DNA cassette in the two strains chrFS (two clones c1 and c2 studied) and chrFS + pCFS (one clone studied). M: DNA size marker 1 kb Plus DNA Ladder, Thermo Scientific GeneRuler. Note the absence of a 2462 bp band corresponding to WT copies of the chromosome lacking the FS gene showing that all chromosome copies possess the *pR*-FS-Km^{R} DNA cassette.

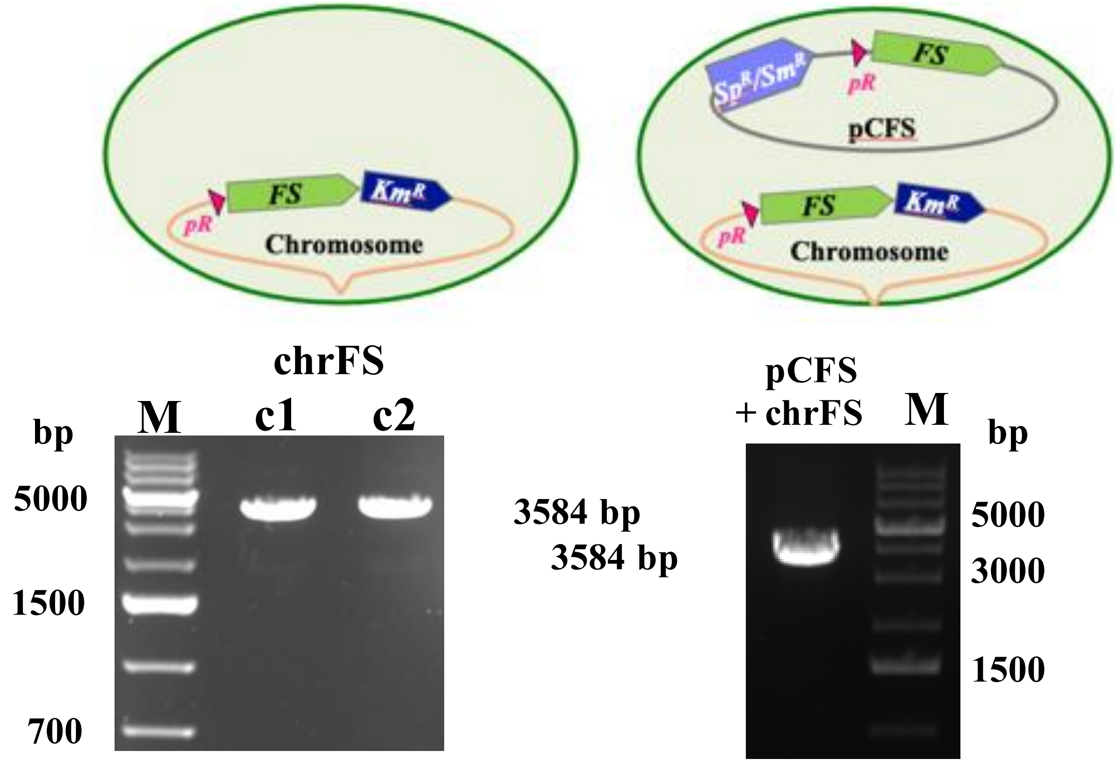

Fig. S2. Influence of dodecane and pC-derived plasmids on the photoautotrophic growth of *Synechocystis*. All strains were cultivated under standard light (2500 lux) and temperature (30°C) in absence (A) or presence (B) of a dodecane overlay: wild-type (**WT**), or its derivatives carrying the **empty pC vector**, or a pC-derived plasmid expressing the limonene synthase (**pCLS**), bisabolene synthase (**pCBS**), farnesene synthase (**pCFS**), pinene synthase (**pCPS**) or santalene synthase (**pCSS**). All experiments were performed at least three times.

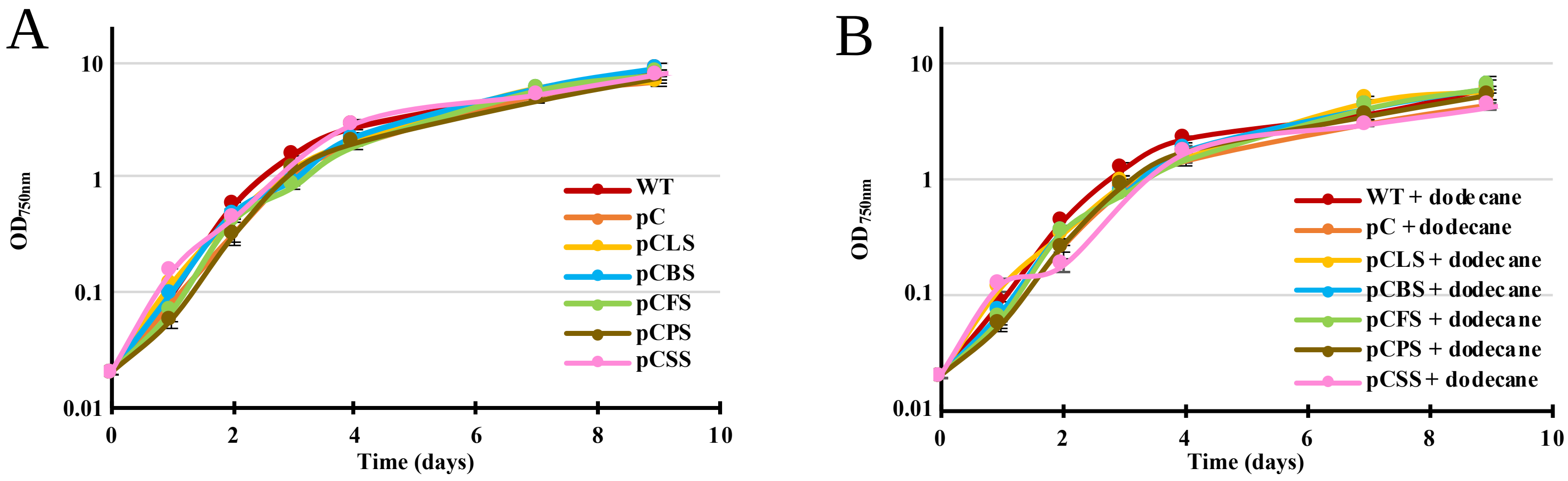

Figure S3. GC–MS analyses of terpene production by the engineered *Synechocystis* strains described in this study.

Fig. S3.A – Standard curve used to calculate the concentration of α-pinene in the dodecane overlay of cultures of the *Synechocystis* strain harboring the pCPS plasmid. Pinene (increasing concentrations) and limonene (internal standard at a fixed concentration) were spiked in dodecane prior to GC-MS analysis.

**Concentration of α-pinene ($mg.L^{-1}$) in dodecane**	**0.1**	**0.3**	**0.5**	**0.8**	**1**	**2.5**	**5**
Area α-pinene	0	244	414	974	573	1330	5860
	0	352	257	666	448	3955	3979
	0	176	399	371	696	1849	1637
Area S-limonene at 10 $mg.L^{-1}$ (IS)	4835	6091	4096	6257	2421	1811	6043
	3908	5811	2502	3921	1901	7131	3396
	2689	3191	4258	2658	3010	3415	1448
Area α-pinene / Area S-limonene	0	0.0400591	0.10107422	0.15566565	0.236679058	0.73440088	0.96971703
	0	0.06057477	0.10271783	0.16985463	0.235665439	0.55462067	1.17167256
	0	0.05515512	0.09370597	0.13957863	0.231229236	0.54143485	1.13052486
**Average**	**0**	**0.05192967**	**0.099166**	**0.15503297**	**0.234524578**	**0.61015213**	**1.09063815**
**Standard deviation**	**0**	**0.01063136**	**0.00479941**	**0.01514791**	**0.002898502**	**0.10780436**	**0.10672264**

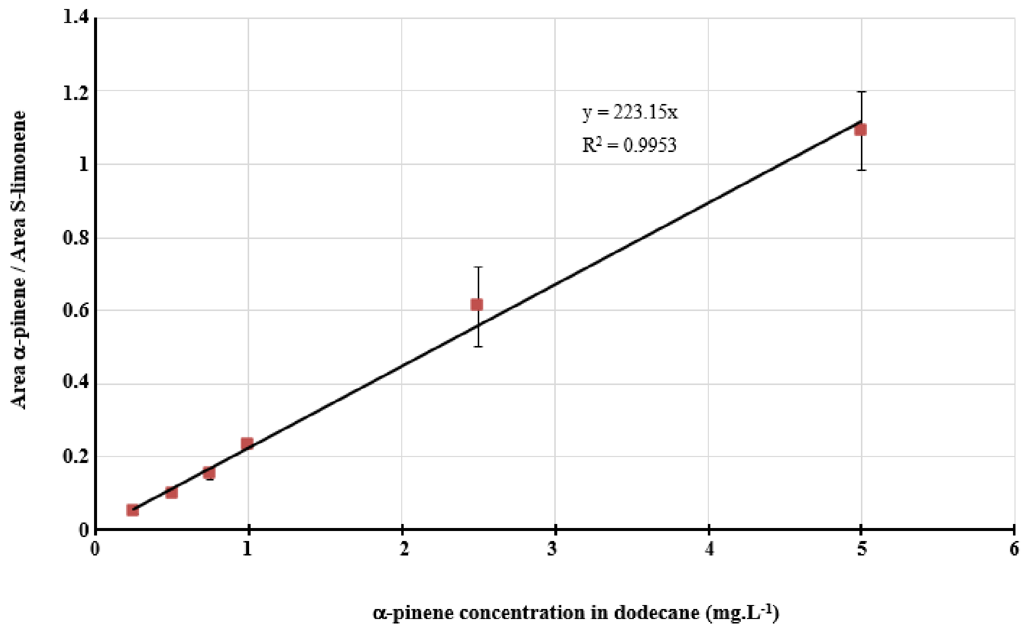

Fig. S3.B. GC–MS analyses of the dodecane overlay of cultures of the *Synechocystis* strains harboring the pC or pCPS plasmids. Ion chromatograms (left panels) and corresponding mass spectra (right panels) of an α-pinene standard (upper panel) or dodecane samples (lower panel) of cultures of *Synechocystis* harboring either pC (negative control) or the pCPS (pinene production). S-(-)-limonene (retention time = 6.89 min) was used as the fixed-concentration (0.01 g.L^{-1}) internal standard (IS) for quantification.

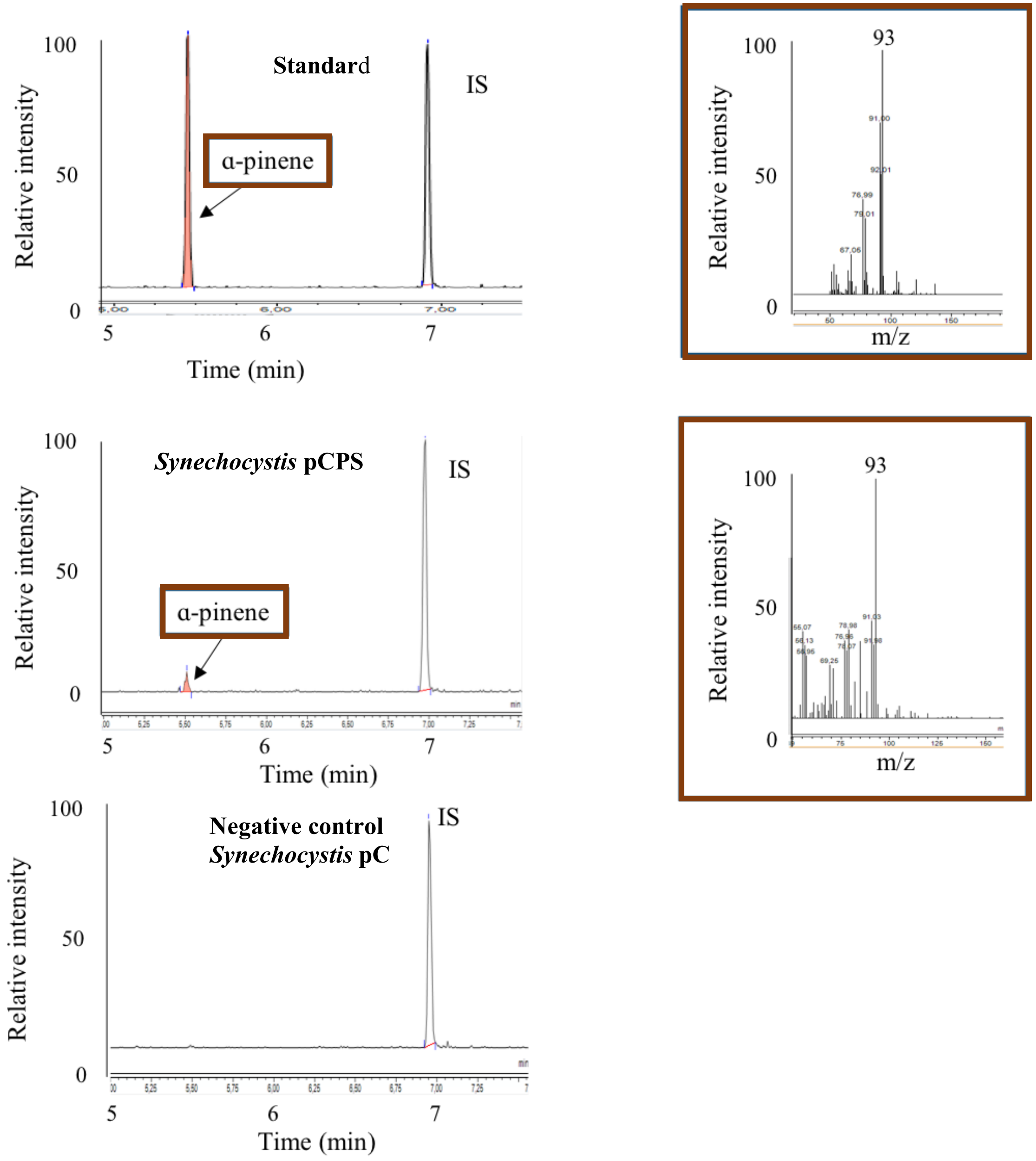

Fig. S3.C. GC–MS analyses of the dodecane overlay of cultures of the *Synechocystis* strains harboring the pC or pCLS plasmids. Ion chromatograms (left panels) and corresponding mass spectra (right panels) of a S-(-)-limonene standard (upper panel) or dodecane samples (lower panel) of cultures of *Synechocystis* harboring either pC (negative control) or the pCLS (S-(-)-limonene production). Pinene (retention time 5.44 min) was used as the fixed-concentration (0.01 g.L^{-1}) internal standard (IS) for quantification. The standard curve used to calculate the concentration of limonene was already published (Chenebault et al., 2020).

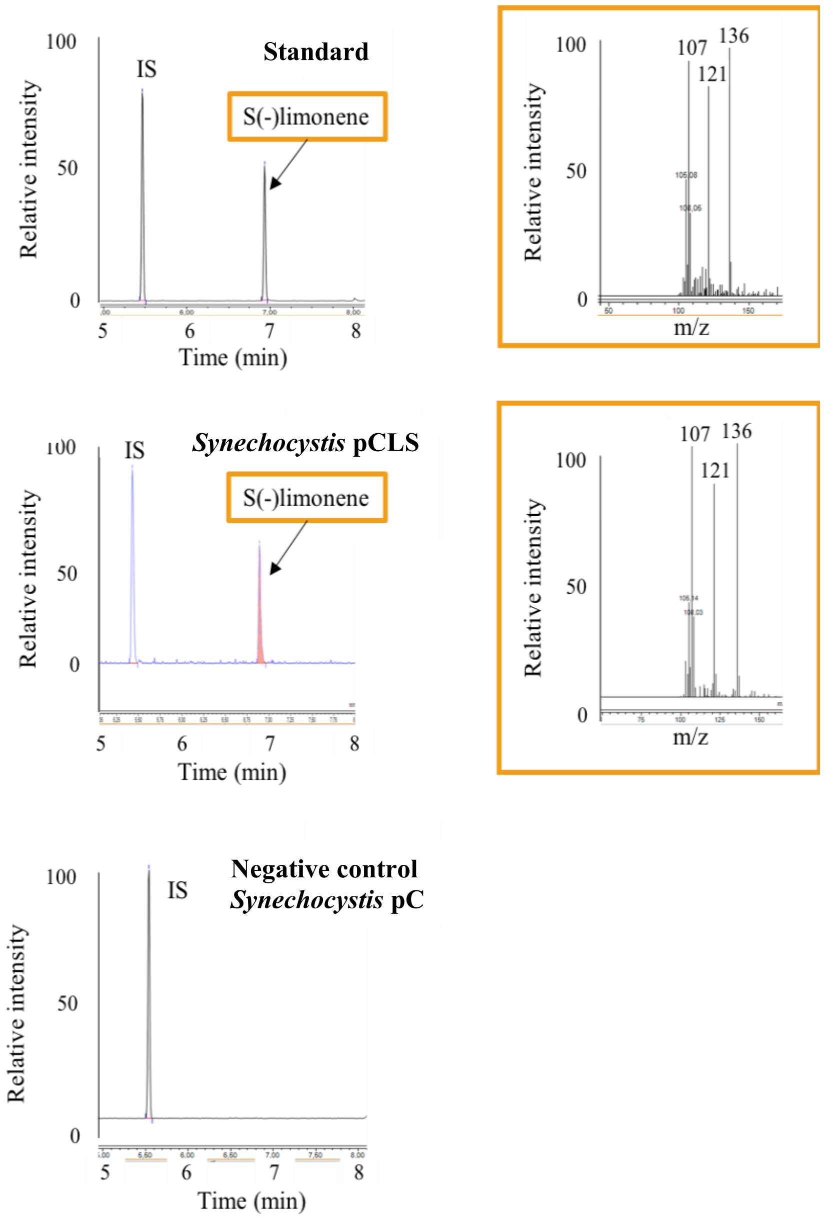

Figure S3.D – Standard curve used to calculate the concentration of E-α-bisabolene in the dodecane overlay of cultures of the *Synechocystis* strain harboring the pCBS plasmid. Bisabolene (increasing concentrations) and trans-caryophyllene (fixed-concentration internal standard) were spiked in dodecane prior to GC-MS analysis. Due to the presence of several bisabolene isomers in the commercial standard, the contribution of (E)-α-bisabolene to the molarity was adjusted based on an attribution of 19.31 ± 0.91% of total combined peak areas to the target molecules, as described (Wichmann et al., 2018).

**Concentration of E-α-bisabolene (mg.L^{-1}) in dodecane**	0.1	0.2	0.5	1	1.4	1.9	3.7	5	7
Area E-α-bisabolene	885	1001	610	6287	4694	8006	6696	6662	21743
	400	0	1892	3660	1999	5990		10223	
	0	238	968	3123	8127	7242		9115	
Area trans-caryophyllene at 10 mg.L^{-1} (IS)	81767	68787	16284	67880	36932	41930	18528	15805	30621
	33649	33070	37689	39086	16876	31490		22942	
	18426	21025	20492	34569	56319	30870		19968	
Area E-α-bisabolene / Area trans-caryophyllene	0.011	0.015	0.037	0.093	0.127	0.191	0.361	0.422	0.710
	0.012	0.000	0.050	0.094	0.118	0.190		0.446	
	0.000	0.011	0.047	0.090	0.144	0.235		0.456	
**Average**	**0.011**	**0.013**	**0.045**	**0.092**	**0.130**	**0.205**	**0.361**	**0.441**	**0.710**
**Standard deviation**	**0.001**	**0.002**	**0.007**	**0.002**	**0.013**	**0.025**		**0.018**	

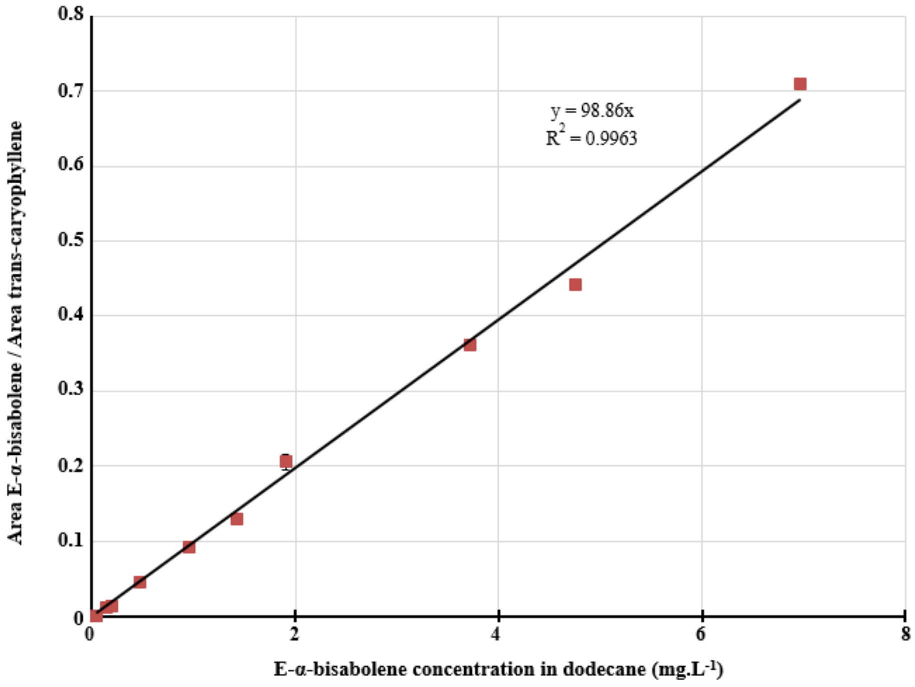

Fig. S3.E GC–MS analyses of the dodecane overlays of cultures of the *Synechocystis* strains harboring the plasmids pC, pCBS or pCFS. Ion chromatograms (left panels) and corresponding mass spectra (right panels) of farnesene-isomers standard (**a**) or dodecane samples from cultures of *Synechocystis* propagating pCFS (**b**, production of α-farnesene), pCBS (**c**, E-α-bisabolene production) or pC (**d**, negative control). β-caryophyllene (retention time = 17.3 min) was used as the fixed-concentration (0.01 g.L^{-1}) internal standard (IS) for quantification.

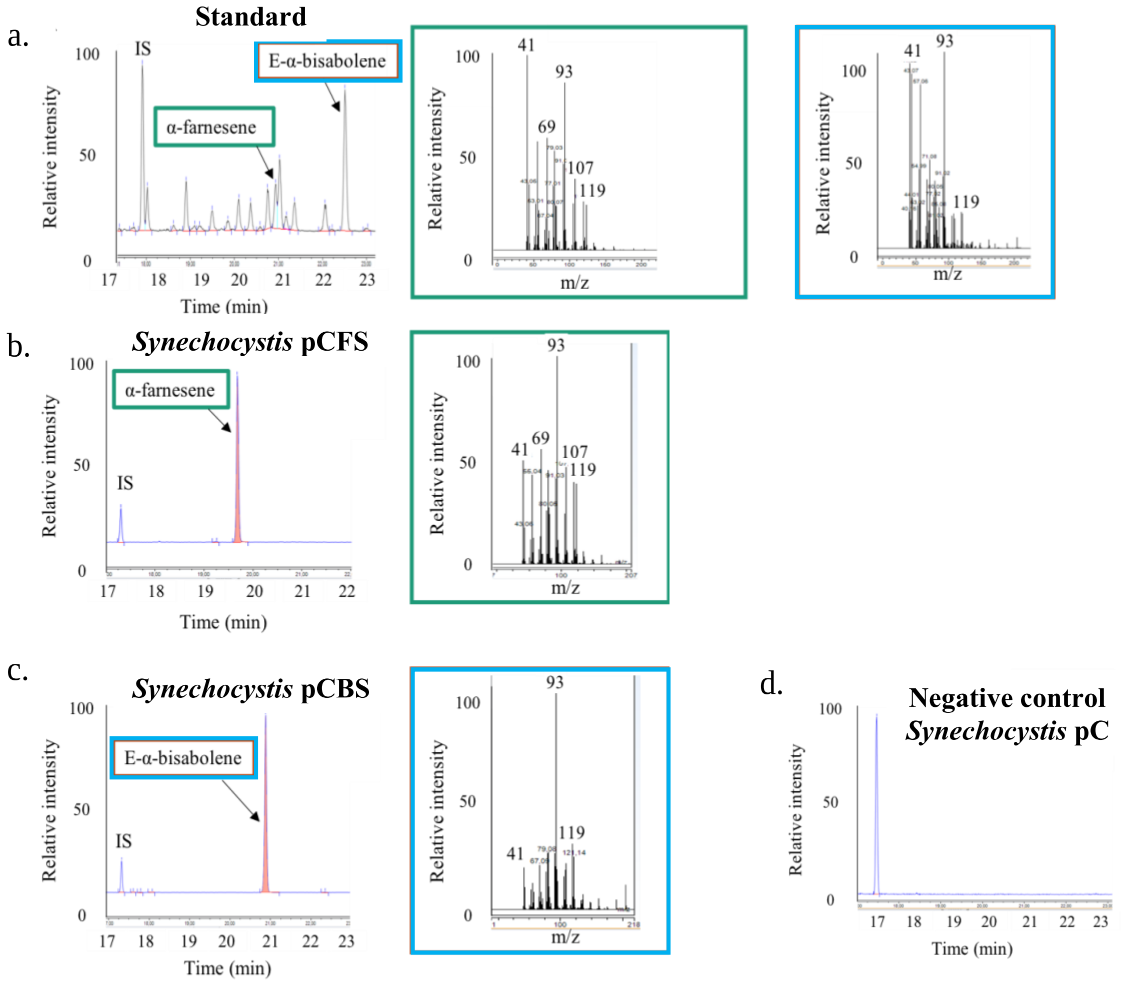

Fig. S3.F. – Standard curve used to calculate the concentration of α-farnesene in the dodecane overlay of cultures of the *Synechocystis* strains harboring the FS gene in either or both the plasmid (pCFS) and the chromosome (chrFS). Farnesene (increasing concentrations) and trans-caryophyllene (fixed-concentration) were spiked in dodecane prior to GC-MS analysis. As the commercial standard contains several farnesene isomers, the contribution of α-farnesene to the molarity was adjusted based on an attribution of 7.78 ± 0.37% of total combined peak areas to the target molecules, as described (Wichmann et al., 2018).

**Concentration of α-farnesene ($mg.L^{-1}$) in dodecane**	0.1	0.2	0.4	0.6	0.8	1.6	2	3
Area α-farnesene	0	0	932	1549	2593	1969	1927	7014
	0	601	532	1719	1689		3057	
	0	273	846	2070	2332		2478	
Area trans-caryophyllene at 10 $mg.L^{-1}$ (IS)	68787	16284	33756	46515	41930	18528	15805	30621
	33070	37689	24305	45600	31490		22942	
	21025	20492	32153	46512	30870		19968	
Area α-farnesene / Area trans-caryophyllene	0	0	0.027	0.033	0.062	0.106	0.121	0.229
	0	0.015	0.021	0.037	0.054		0.133	
	0	0.013	0.026	0.044	0.076		0.124	
**Average**	**0**	**0.0146**	**0.0252**	**0.0385**	**0.0636**	**0.106**	**0.126**	**0.229**
**Standard deviation**	**0**	**0.0018**	**0.0030**	**0.0056**	**0.0110**		**0.0067**	

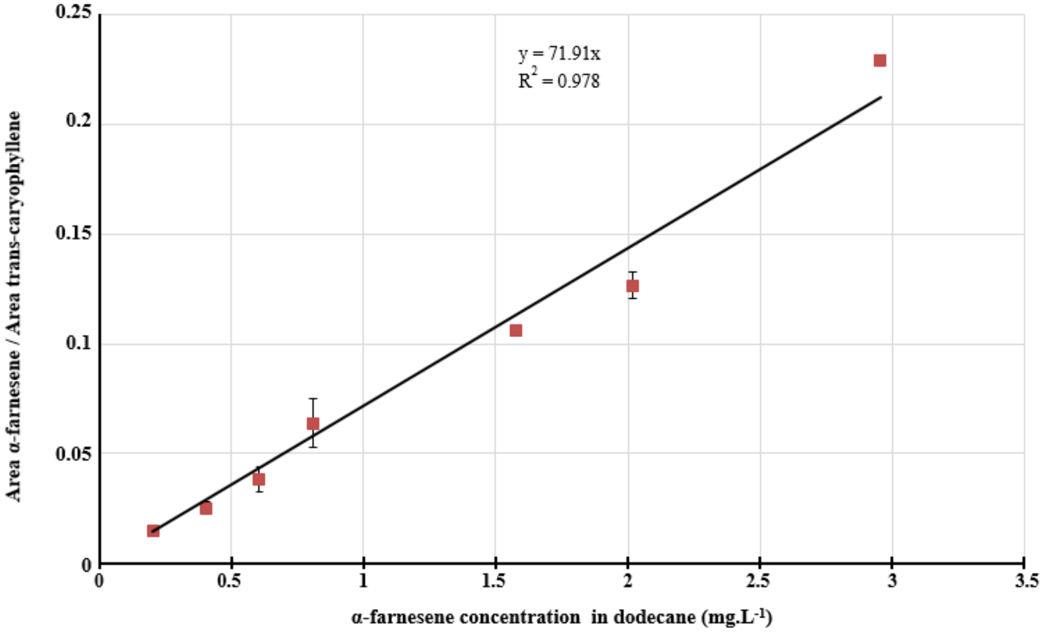

Figure S3.G. Standard curve used to calculate the concentration of α-santalene in the dodecane overlay of cultures of the *Synechocystis* strain harboring the pCSS plasmid. α-santalene (increasing concentrations) and nerolidol (fixed-concentration) were spiked in dodecane prior to GC-MS analysis. As the commercial standard contains several santalene, bergamotene, santalol and bergamotol isomers, the contribution of α-santalene to the molarity was adjusted based on an attribution of only 0.27±0.02% of total combined peak areas to the target molecules, as described (Wichmann et al., 2018).

**Concentration of α-santalene ($mg.L^{-1}$) in dodecane**	**0.07**	**0.13**	**0.27**	**0.53**	**1.33**	**2.00**	**2.67**	**4.00**
Area α-santalene	**0**	1621	360	1432	2848	4140	3443	4797
	947	1494	446	744	1671	4447	3497	4734
		363	395	755	2151	2444	5281	3671
Area nerolidol at 10 $mg.L^{-1}$ (IS) (area cis-nerolidol + trans-nerolidol)	43133	55945	3891	7765	5943	6569	3561	3548
	48969	48677	6699	3369	4260	7206	3915	3451
		7268	4685	4571	5422	4056	6914	3425
Area α-santalene / Area nerolidol	0	0.029	0.093	0.18	0.48	0.63	0.97	1.35
	0.019	0.031	0.067	0.22	0.39	0.62	0.89	1.37
		0.050	0.084	0.17	0.40	0.60	0.76	1.07
**Average**	**0.01**	**0.04**	**0.08**	**0.19**	**0.42**	**0.62**	**0.87**	**1.27**
**Standard deviation**	**0.014**	**0.014**	**0.013**	**0.028**	**0.049**	**0.014**	**0.103**	**0.168**

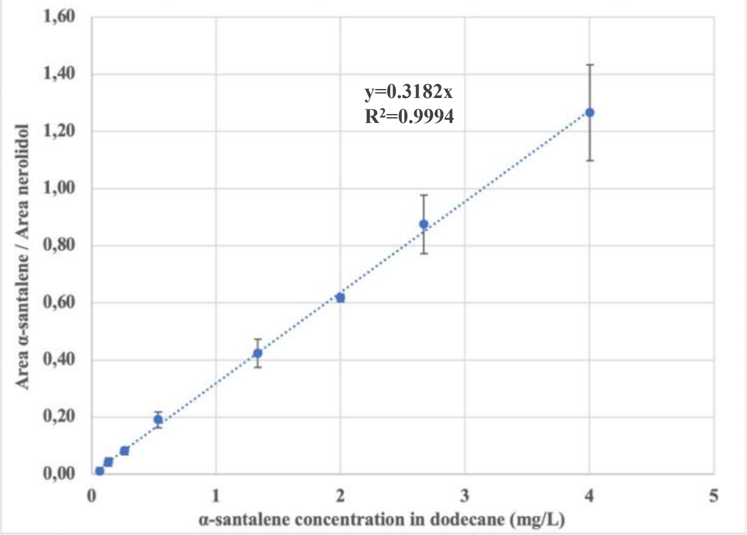

Figure S3.H – Standard curve used to calculate the concentration of α-exo-bergamotene in the dodecane overlay of cultures of the *Synechocystis* strain harboring pCSS. α-exo-bergamotene (increasing concentrations) and nerolidol (fixed-concentration internal standard) were spiked in dodecane prior to GC-MS analysis. As the commercial standard contains several santalene, bergamotene, santalol and bergamotol isomers, the contribution of α-exo-bergamotene to the molarity was adjusted based on an attribution of only 0.1% of total combined peak areas to the target molecules, as described (Wichmann et al., 2018).

**Concentration of α-exo-bergamotene (mg.L^{-1}) in dodecane**	0.075	0.150	0.375	0.563	0.750
Area α-exo-bergamotene	0	824	1669	2392	1915
	411	561	1061	2156	1682
	0	504	1061	1307	2635
Area nerolidol at 10 mg.L^{-1} (IS)	3891	7765	5943	6569	3561
	6699	3369	2972	7206	3915
	4685	4571	5422	4056	6914
Area α-exo-bergamotene / Area nerolidol	0.000	0.106	0.281	0.364	0.538
	0.061	0.167	0.249	0.299	0.430
	0.000	0.110	0.196	0.322	0.381
**Average**	**0.061**	**0.128**	**0.242**	**0.329**	**0.450**
**Standard deviation**	**0.000**	**0.034**	**0.043**	**0.033**	**0.080**

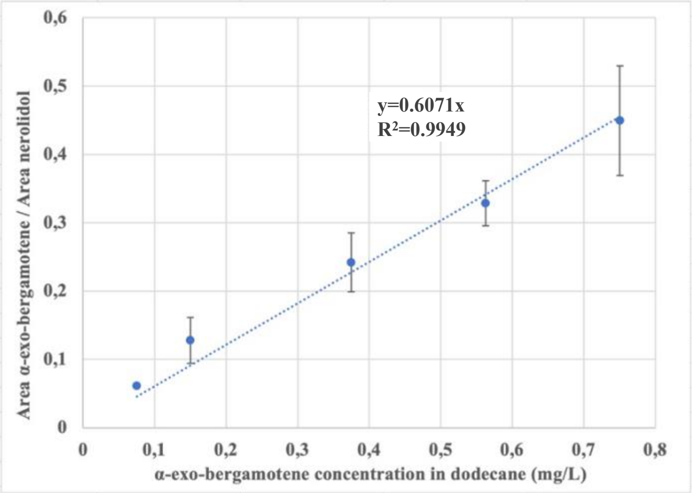

Figure S3.I. – Standard curve used to calculate the concentration of epi-β-santalene in the dodecane overlay of cultures of the *Synechocystis* strain harboring pCSS. epi-β-santalene (increasing concentrations) and nerolidol (fixed-concentration internal standard) were spiked in dodecane prior to GC-MS analysis. As the commercial standard contains several santalene, bergamotene, santalol and bergamotol isomers, the contribution of epi-β-santalene to the molarity was adjusted based on an attribution of only 0.39%±0.02% of total combined peak areas to the target molecules, as described (Wichmann et al., 2018).

**Concentration of epi-β-santalene ($mg.L^{-1}$) in dodecane**	0.20	0.39	0.78	1.95	2.93	3.91	5.86	7.82
Area epi-β-santalene	1592	281	1125	2142	3669	2975	4214	8173
	1025	372	630	1453	4185	2852	3909	8012
	327	321	763	1893	2091	4833	3223	8173
Area nerolidol at 10 $mg.L^{-1}$ (IS)	55945	3891	7765	5943	6569	3561	3548	6529
	48677	6699	3369	4260	4646	3915	3451	6559
	7268	4685	4571	5422	4056	6914	3425	6529
Area epi-β-santalene / Area nerolidol	0.03	0.07	0.14	0.36	0.56	0.84	1.19	1.25
	0.03	0.06	0.19	0.34	0.58	0.73	1.13	1.22
	0.04	0.07	0.17	0.35	0.52	0.70	0.94	1.43
**Average**	**0.032**	**0.065**	**0.166**	**0.350**	**0.553**	**0.754**	**1.087**	**1.300**
**Standard deviation**	**0.012**	**0.009**	**0.021**	**0.010**	**0.034**	**0.072**	**0.130**	**0.111**

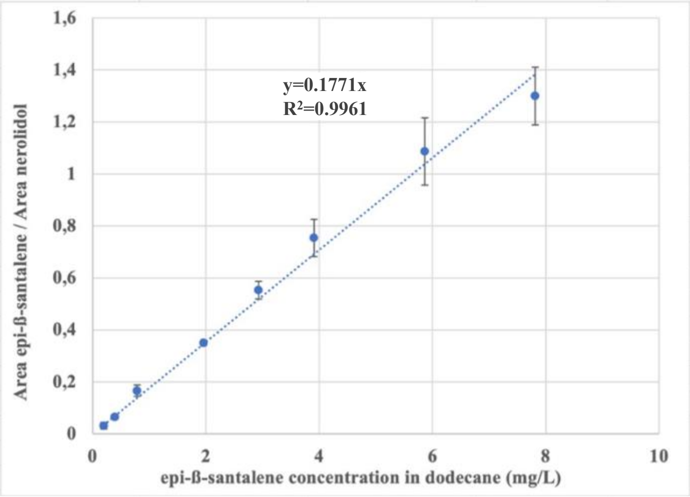

Figure S3.J. GC–MS analyses of the dodecane overlays of cultures of the *Synechocystis* strain harboring the pCSS plasmid. m/z=93 ion chromatograms (upper panel) and mass spectra (lower panels) of the α-santalene (framed in mauve), epi-β-santalene (framed in purple) and α-exo-bergamotene (framed in pink) isomers were obtained from GC-MS analyses of a *Synechocystis* pCSS culture grown for 21 days. Nerolidol at 0.01 g.L^{-1} was used as the internal standard (IS) for quantification.

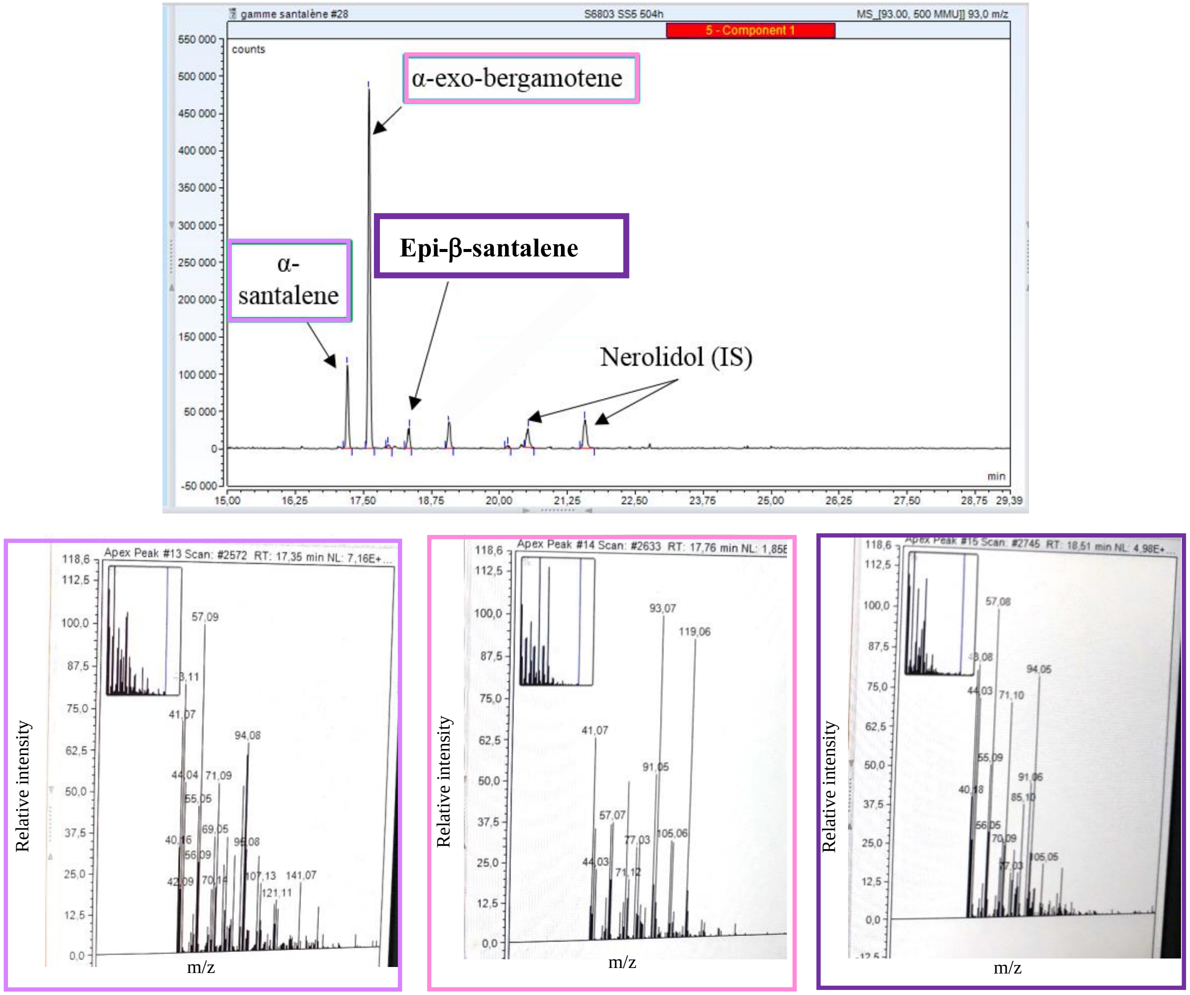

Figure S3.K. Comparison of the photosynthetic production of terpenes by the engineered *Synechocystis* strains constructed in this study.

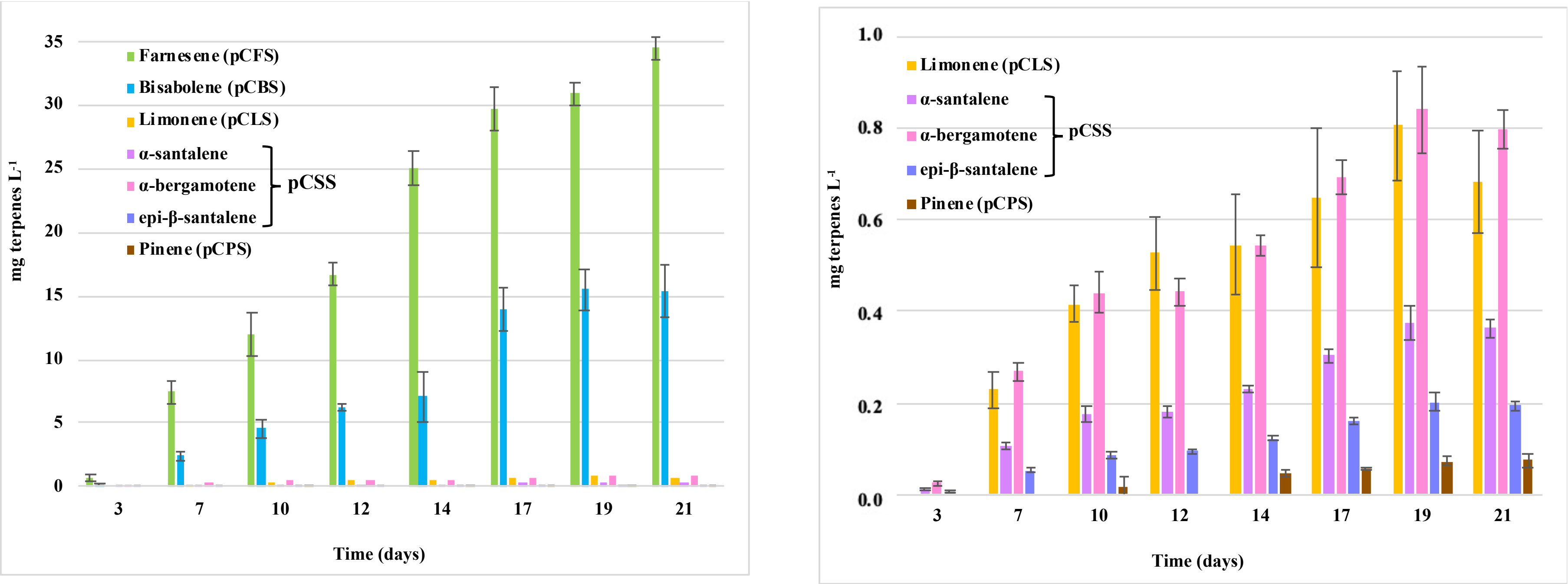

Supplement: Supplementary file 1 — Additional file 1. Additional tables and figures. [file 13068_2022_2211_MOESM1_ESM.pdf]
